# *Pseudomonas aeruginosa* AlgR Phosphorylation Status Differentially Regulates Pyocyanin and Pyoverdine Production

**DOI:** 10.1128/mBio.02318-17

**Published:** 2018-01-30

**Authors:** Alexander S. Little, Yuta Okkotsu, Alexandria A. Reinhart, F. Heath Damron, Mariette Barbier, Brandon Barrett, Amanda G. Oglesby-Sherrouse, Joanna B. Goldberg, William L. Cody, Michael J. Schurr, Michael L. Vasil, Michael J. Schurr

**Affiliations:** a Department of Immunology and Microbiology, University of Colorado School of Medicine, Aurora, Colorado, USA; b Department of Pharmaceutical Sciences, University of Maryland School of Pharmacy, Baltimore, Maryland, USA; c Department of Microbiology and Immunology, University of Maryland School of Medicine, Baltimore, Maryland, USA; d Department of Microbiology, Immunology, and Cancer Biology, University of Virginia School of Medicine, Charlottesville, Virginia, USA; e Department of Microbiology, Immunology, and Cell Biology, West Virginia University, Morgantown, West Virginia, USA; f Department of Biology, University of Dallas, Irving, Texas, USA; g Department of Pediatrics, Emory University School of Medicine, Atlanta, Georgia, USA; h Center for Cystic Fibrosis and Airways Disease Research, Children’s Healthcare of Atlanta, Atlanta, Georgia, USA; i Department of Surgery, University of Colorado School of Medicine, Aurora, Colorado, USA; Harvard Medical School

**Keywords:** AlgR, *Pseudomonas aeruginosa*, iron acquisition, pyocyanin, pyoverdine, virulence regulation

## Abstract

*Pseudomonas aeruginosa* employs numerous, complex regulatory elements to control expression of its many virulence systems. The *P. aeruginosa* AlgZR two-component regulatory system controls the expression of several crucial virulence phenotypes. We recently determined, through transcriptomic profiling of a PAO1 Δ*algR* mutant strain compared to wild-type PAO1, that *algZR* and *hemCD* are cotranscribed and show differential iron-dependent gene expression. Previous expression profiling was performed in strains without *algR* and revealed that AlgR acts as either an activator or repressor, depending on the gene. Thus, examination of *P. aeruginosa* gene expression from cells locked into different AlgR phosphorylation states reveals greater physiological relevance. Therefore, gene expression from strains carrying *algR* alleles encoding a phosphomimetic (AlgR D54E) or a phosphoablative (AlgR D54N) form were compared by microarray to PAO1. Transcriptome analyses of these strains revealed 25 differentially expressed genes associated with iron siderophore biosynthesis or heme acquisition or production. The PAO1 *algR D54N* mutant produced lower levels of pyoverdine but increased expression of the small RNAs *prrf1* and *prrf2* compared to PAO1. In contrast, the *algR D54N* mutant produced more pyocyanin than wild-type PAO1. On the other hand, the PAO1 *algR D54E* mutant produced higher levels of pyoverdine, likely due to increased expression of an iron-regulated gene encoding the sigma factor *pvdS*, but it had decreased pyocyanin production. AlgR specifically bound to the *prrf2* and *pvdS* promoters *in vitro*. AlgR-dependent pyoverdine production was additionally influenced by carbon source rather than the extracellular iron concentration *per se*. AlgR phosphorylation effects were also examined in a *Drosophila melanogaster* feeding, murine acute pneumonia, and punch wound infection models. Abrogation of AlgR phosphorylation attenuated *P. aeruginosa* virulence in these infection models. These results show that the AlgR phosphorylation state can directly, as well as indirectly, modulate the expression of iron acquisition genes that may ultimately impact the ability of *P. aeruginosa* to establish and maintain an infection.

## IMPORTANCE

Pyoverdine and pyocyanin production are well-known *P. aeruginosa* virulence factors that obtain extracellular iron from the environment and from host proteins in different manners. Here, we show that the AlgR phosphorylation state inversely controls pyoverdine and pyocyanin production and that this control is carbon source dependent. *P. aeruginosa* expressing AlgR D54N, mimicking the constitutively unphosphorylated state, produced more pyocyanin than cells expressing wild-type AlgR. In contrast, a strain expressing an AlgR phosphomimetic (AlgR D54E) produced higher levels of pyoverdine. Pyoverdine production was directly controlled through the *prrf2* small regulatory RNA and the pyoverdine sigma factor, PvdS. Abrogating pyoverdine or pyocyanin gene expression has been shown to attenuate virulence in a variety of models. Moreover, the inability to phosphorylate AlgR attenuates virulence in three different models, a *Drosophila melanogaster* feeding model, a murine acute pneumonia model, and a wound infection model. Interestingly, AlgR-dependent pyoverdine production was responsive to carbon source, indicating that this regulation has additional complexities that merit further study.

## INTRODUCTION

*Pseudomonas aeruginosa* is a significant pathogen in cystic fibrosis (CF) patients, in whom colonization in the lung is linked to a worsening disease prognosis (1, 2). It is also a significant causative agent of nosocomial infections (3–7), particularly in burn wounds (8, 9) and immunocompromised individuals (10–12). There are several different virulence factors which *P. aeruginosa* utilizes to cause infection in different hosts, and expression of many of these respond to low iron, including alginate and toxin production (13).

*P. aeruginosa* utilizes at least 64 sensor kinases and 73 response regulators to control these systems and to sense its environment, and these sensors result in altered gene expression for optimal survival (14, 15). Two-component systems frequently employ a membrane-bound histidine kinase that perceives an environmental signal and transduces that signal to a DNA binding response regulatory protein through phosphorylation, which alters its affinity for DNA (16, 17). The *P. aeruginosa* AlgZR two-component regulatory system is composed of a response regulator, AlgR (18–22), and a putative histidine kinase, named AlgZ (23) or FimS (24). AlgR was initially described as a response regulator required for alginate production via control of the *algD* and *algC* promoters (18–20, 25, 26). The functions of the AlgR regulon have gradually expanded to include twitching motility (21, 24, 27), cyanide production (21, 28, 29), as well as rhamnolipid production (30, 31). In fact, the AlgZR regulon was recently characterized via chromatin immunoprecipitation sequencing (ChIP-seq) to include 157 AlgR binding sites associated with 155 genes (32). The majority of the identified AlgR binding sites are associated with genes involved in carbon metabolism. However, because these experiments relied on the overexpression of AlgR, there remains the possibility of false-positive binding sites. Confirmatory expression data obtained by using reverse transcription-quantitative PCR (RT-qPCR) and DNA binding assays were included for only a few genes in that study; it remains to be determined how many of these genes are directly regulated by AlgR.

The PAO1 and mutant strain PAO1 Δ*algR* gene expression profiles from logarithmic, stationary, and biofilm growth conditions (21, 30) revealed the differential regulation of several iron- or heme related-genes, including, *hemN*, *katB*, *ccoP2*, *ccoQ2*, *ccoN2*, and *ccoO2* (21). Phenazine gene expression (e.g., *phzC2-phzG2*) was also increased in a biofilm formed by strain PAO1 Δ*algR* (30). More recently, additional links between iron and alginate regulation have been observed, including that alginate production responds inversely to iron concentration (33) and that *algZR* is cotranscribed with *hemCD*, which encodes the enzymes porphobilinogen deaminase and uroporphyrinogen III synthetase, which are involved in the second and third steps in heme biosynthesis (34, 35). Lastly, a study by Kong et al. identified an AlgR binding site within the regulatory region of the Fur-regulated small RNA (sRNA) *prrf2*, as well as those of other genes encoding iron-containing proteins (e.g., aconitase and fumarase) (32). Altogether, these data indicate that AlgR likely controls iron-related gene expression and genes encoding iron-containing proteins.

Multiple studies have tied together the AlgZR system with virulence in *P. aeruginosa* (32, 36–38). *P. aeruginosa* murine septicemia was attenuated when *algR* was deleted, with 74% survival compared to 0% with PAO1, but interestingly, the mutant strain was even more attenuated, resulting in 100% survival when *algR* was overexpressed (36). Coinfection using a pneumonia model with both PAO1 and strain PAO1 Δ*algR* showed that strain PAO1Δ*algR* was cleared more rapidly from the murine lung (36). Interestingly, recent data from a murine pneumonia model showed that while strain PAO1 Δ*algR* was attenuated for virulence by delayed killing relative to the parental strain, strain PAO1 Δ*algZ* was even more highly attenuated, with 60% murine survival over the course of the 5-day experiment (32). These findings suggest that while there are ample available data on the virulence effects of deleting *algR*, the phosphorylation state of AlgR may be the most important aspect in terms of virulence.

Here, we show that AlgR regulates a variety of iron-associated genes and indirectly controls pyocyanin production; it also directly and indirectly controls pyoverdine production through its phosphorylation state. The phosphorylation state has been mimicked for AlgR, including use of mutant strains PAO1 *algR D54N* and PAO1 *algR D54E*, resulting in constitutive unphosphorylation and phosphorylation, respectively. Functional characterization of these mutants largely focused on expression of functional type IV pili and their inability to be subsequently phosphorylated by a heterologous kinase, CheA (27, 31). Phosphorylation of AlgR by the predicted cognate histidine kinase AlgZ has not been shown biochemically, but only genetically inferred (23, 29). Mutant strain PAO1 *algR D54N*, encoding unphosphorylated AlgR, exerted a repressive effect on pyoverdine production, in contrast to the mutant PAO1 strain that expressed the AlgR phosphomimetic mutant (AlgR D54E) that results in increased pyoverdine production. AlgR-repressed pyoverdine production occurs through two mechanisms: (i) by activating *prrf2* small regulatory RNA expression and (ii) direct repression of *pvdS* expression, which encodes the sigma factor required for pyoverdine production. Interestingly, iron concentrations over 25 µM suppressed the AlgR phosphorylation effect on pyoverdine production, suggesting that there is a hierarchy for iron acquisition gene expression. Moreover, the AlgR phosphorylation-dependent iron effects were reversible with different carbon sources. The AlgR phosphorylation state was extremely important in multiple virulence models, as the phosphoablated strain (PAO1 *algR D54N*) was attenuated in a *Drosophila* feeding model of infection and in acute murine wound and pneumonia models of infection.

## RESULTS

### *fimU* expression coincides with *pvdS* expression and siderophore production.

The *fimUpilVWXY1Y2E* operon requires phosphorylated AlgR for expression (21, 24, 27, 39); therefore, *fimU* expression was determined using RT-qPCR as a measure of AlgR phosphorylation through the growth curve (see Fig. S1A in the supplemental material). PAO1 *fimU* expression was low through the logarithmic growth phase but sharply increased as the cells entered stationary phase, indicating that AlgR activity was maximal in stationary phase under the conditions tested. Since *fimU* expression was maximal in late stationary phase, strongly indicating when AlgR is phosphorylated in the growth phase, strains previously constructed that contained either the phosphomimetic (*algR D54E*) allele (31) or the phosphoablated (*algR D54N*) allele (27) were compared by transcriptomic analysis to the wild-type strain PAO1 at late stationary phase in Miller LB medium. Our Miller LB formulation contained 6 µM total iron, as measured using ferrozine (data not shown). Comparison of the PAO1 transcriptome to mutant strain PAO1 *algR D54E* and PAO1 *algR D54N* transcriptomes revealed a large number of differentially expressed genes, including those involved in transport of small molecules, membrane proteins, transcriptional and translational processing, and transcriptional regulators (Table S2, parts B to D). The transcriptome of PAO1 compared to those of strains PAO1 *algR D54N* and PAO1 *algR D54E* revealed 68 and 5 statistically significant, differentially regulated genes, respectively (Table S2, parts B and C). There were 154 differentially and statistically significant differently expressed genes when global expression in strain PAO1 *algR D54N* was compared to that of strain PAO1 *algR D54E* (Table S2, part D). The comparison of strains PAO1 and PAO1 *algR D54E* showed that only 5 genes were differentially regulated, indicating that PAO1 gene expression in late stationary phase was almost identical to that for PAO1 *algR D54E* and further suggested that AlgR was predominantly phosphorylated under this condition. These results were also consistent with maximal *fimU* expression in late stationary phase (Fig. S1A). Some of the most highly differentially expressed genes in strain PAO1 *algR D54N* (compared to PAO1) were related to siderophore production and iron or heme acquisition (Table S2, parts B and D). All of these iron-, heme-, or siderophore-related genes were more highly expressed in strains PAO1 and PAO1 *algR D54E* than in strain PAO1 *algR D54N* (Table S2). These data indicate that AlgR phosphorylation results in the activation or derepression of these target genes. The 25 iron-related genes that were differentially expressed included 5 genes associated with heme uptake, 10 genes associated with iron uptake or an iron starvation response, and 10 genes associated with pyoverdine or pyochelin production or uptake of xenosiderophores (Table S2, part A).

10.1128/mBio.02318-17.2 FIG S1  Influence of time on *fimU*, *pvdS*, and siderophore expression. RT-qPCR expression of *fimU* (A), pyoverdine production (representative data set, red bars, left *y* axis) (B), and RT-qPCR expression of *pvdS* (black bars, left *y* axis), graphed along with the increasing optical density (OD_600_) of the PAO1 culture (line, right *y* axis) (C). Data were collected at time points between 6.5 and 16 h. Download FIG S1, TIF file, 21 MB. Copyright © 2018 Little et al. 2018 Little et al. This content is distributed under the terms of the Creative Commons Attribution 4.0 International license.

Since 10 different pyoverdine-associated genes were found to be differentially expressed in the transcriptomic analyses, the relationship between AlgR phosphorylation state and pyoverdine production was determined throughout the same growth curve by following excretion of pyoverdine into the culture medium (Fig. S1B). Like *fimU* expression, pyoverdine production increased over time, into stationary phase. Since expression of pyoverdine genes is predominantly mediated by the sigma factor *pvdS*, expression of *pvdS* was measured by RT-qPCR throughout growth (Fig. S1C). Similar to both *fimU* expression and pyoverdine production itself, expression of *pvdS* was higher at later points in growth. These results indicated that there was a correlation between *pvdS* expression, siderophore production, and *fimU* expression and that late stationary phase was when maximal AlgR phosphorylation-dependent gene expression occurred. Therefore, these conditions were used to examine AlgR-dependent siderophore production and whether PAO1 *algR D54E* and PAO1 *algR D54N* could be predicted to produce different amounts of siderophores.

### Expression of *algR* D54N repressed pyocyanin production.

**FIG 1:**
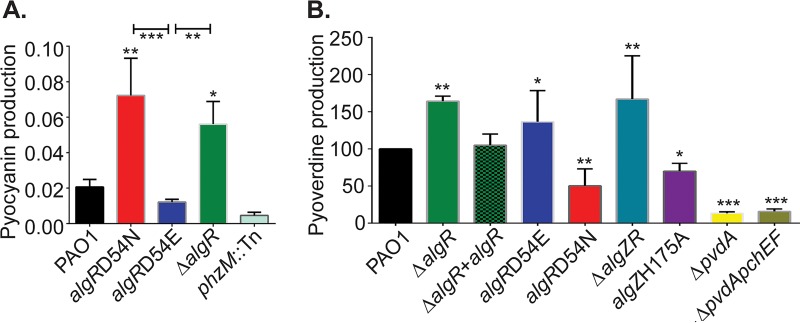
Effect of *algR* mutations on pyocyanin and pyoverdine production. (A) Measure of pyocyanin production between PAO1 (wild type) and the strain PAO1 *algR* mutants PAO1 *algR D54N*, PAO1 *algR* D54E, and PAO1 Δ*algR*, compared to a PAO1 *phzM*::Tn negative control. (B) Iron chelation of isolated supernatants in liquid CAS medium, relative to results with PAO1, using the strains in panel A and the PAO1 *algR* complementation strain PAO1Δ*algR* + pRCM-7, PAO1 Δ*algZR*, PAO1 *algZ H175A*, PAO1 Δ*pvdA*, and PAO1 Δ*pvdA* Δ*pchEF*. Data were analyzed by ANOVA with the Bonferroni multiple-comparison test. Asterisks denote comparison to PAO1, except where noted by a bar. *, *P* < 0.05; **, *P* < 0.01; ***, *P* < 0.001.

Previous microarray data comparing strain PAO1 to PAO1 Δ*algR* under biofilm growth conditions identified *phzD2*, *phzE2*, *phzF2*, and *phzG2* (PA1902 to PA1905) as AlgR regulated (30). Kong et al. showed that *algR* deletion resulted in increased pyocyanin production through *czcC* and *czcR* regulation, a system generally involved in heavy metal and carbepenem resistance (32, 40, 41). In the Kong study, the effect of *algR* deletion was examined, but the AlgR phosphorylation state that led to this regulation was not determined (32). In order to determine the effects of AlgR phosphorylation on pyocyanin production, strains PAO1, PAO1 Δ*algR*, PAO1 *algR D54N*, and PAO1 *algR D54E* were compared. When pyocyanin production was directly measured from liquid medium, both PAO1 *algR* D54N and PAO1 Δ*algR* produced considerably more (3.6× and 2.8×, respectively) pyocyanin than the wild type (Fig. 1A). Strain PAO1 *algR D54E* produced considerably less pyocyanin than either mutant strain, PAO1 *algR D54N* or PAO1 Δ*algR*, but not compared to the wild type (Fig. 1A). To further investigate the effects of AlgR phosphorylation on pyocyanin production, plasmids carrying either wild-type *algR*, *algR D54N*, or *algR D54E* were transferred into the PAO1 Δ*algR* background and pyocyanin production was measured. Increased *algR D54N* expression did not alter pyocyanin production but induced expression of wild-type *algR* or *algR D54E-*repressed pyocyanin production (3× and 3.2×, respectively) (Fig. S2A). Very similar results were obtained when these strains were grown in King’s B medium, indicating that the AlgR-dependent pyocyanin control was not medium dependent (Fig. S2B).

10.1128/mBio.02318-17.3 FIG S2  Effect of *algR* mutations on pyocyanin production. Measurements of pyocyanin production with complementation of *algR* forms back into the PAO1 Δ*algR* background in LB medium (A) or King’s B medium (B) by using pHERD30T containing wild-type *algR* (black), AlgR D54N (red), or AlgR D54E (blue). Data were analyzed by ANOVA with a Bonferroni multiple-comparison test. Asterisks denote comparison to PAO1: *, *P* < 0.05; **, *P* < 0.01; ***, *P* < 0.001. Download FIG S2, TIF file, 14.2 MB. Copyright © 2018 Little et al. 2018 Little et al. This content is distributed under the terms of the Creative Commons Attribution 4.0 International license.

### AlgR controls pyoverdine production.

Comparison of the mutant strain PAO1 *algR D54E* and PAO1 *algR D54N* transcriptomes revealed that four pyoverdine biosynthetic genes were statistically differentially expressed (*pvdF*, *pvdD*, *pvdI*, and *pvdX*) (Table S2, part A). To further investigate this, the pyoverdine fluorescence (based on the optical density at 405 nm [OD_405_]) was measured from supernatants of strains PAO1, PAO1 *algR D54N*, PAO1 *algR D54E*, and PAO1 Δ*algR*. The *algR* mutant strain supernatants showed major differences compared to their isogenic wild-type strain. Both strains PAO1 *algR D54E* and PAO1 Δ*algR* had twice (2.1× and 2.6×, respectively) the fluorescence of PAO1, while strain PAO1 *algR D54N* had less than half (0.45×) the fluorescence of the wild type (Fig. S3A). Since the pyoverdine and pyochelin fluorescent spectra overlap (42), the supernatants from broth-grown cultures were examined by using a previously described liquid chrome-azurol-S (CAS) assay (43). The strains PAO1, PAO1 Δ*algR*, PAO1 *algR D54E*, PAO1 *algR D54N*, PAO1 *algZ H175A*, PAO1 Δ*algZR*, and the siderophore-defective mutants PAO1 Δ*pvdA* and PAO1 Δ*pvdA* Δ*pchEF* were examined. Strain PAO1 *algZ H175A* was created to determine if an inactivated AlgZ would phenocopy PAO1 *algR D54N*. The AlgZ histidine 175, which is predicted to be autophosphorylated if the protein is a histidine kinase, was replaced with an alanine and placed onto the PAO1 chromosome. This strain, PAO1 *algZ H175A*, is unable to perform type IV pilus-mediated twitching motility, a result consistent with the inability of the encoded protein to be autophosphorylated (Fig. S4). Strains PAO1 *algR D54N* and PAO1 *algZ H175A* produced significantly fewer siderophores than the parental PAO1 strain (Fig. 1B). The strains PAO1 Δ*algR*, PAO1 *algR D54E*, and PAO1 Δ*algZR* produced considerably more siderophores than the wild type, consistent with the previous fluorescense data. These data also indicate that the CAS assay only detects pyoverdine production, and not pyochelin production, in LB supernatants, since no iron-chelating capacity was detected from strain PAO1 Δ*pvdA* supernatants and there was no change in siderophore detection between strains PAO1 Δ*pvdA* and PAO1 Δ*pvdA* Δ*pchEF* (Fig. 1B).

10.1128/mBio.02318-17.4 FIG S3  Effect of AlgR phosphorylation on optimal pyoverdine production. (A) Measurements of pyoverdine production based on OD_405_ fluorescence (using strains described for Fig. 1). (B) Iron chelation of isolated supernatants from the related and mucoid *P. aeruginosa* strains, in liquid CAS medium, relative to their parental strain. (Parental strains are shown in black/gray shades, and Δ*algR* derivatives are shown in greens). (C) Strain PAO1 Δ*algR* containing either AlgR D54E (blue) or AlgR D54N (red) on an arabinose-inducible plasmid were compared relative to PAO1Δ*algR* containing an empty vector (green) for iron chelation of isolated supernatants in liquid CAS medium. Data were analyzed by ANOVA with a Bonferroni multiple-comparison test. Asterisks denote comparison to the parental strain: *, *P* < 0.05; **, *P* < 0.01; ***, *P* < 0.001. Download FIG S3, TIF file, 31.2 MB. Copyright © 2018 Little et al. 2018 Little et al. This content is distributed under the terms of the Creative Commons Attribution 4.0 International license.

10.1128/mBio.02318-17.5 FIG S4  Strain PAO1 *algZ H175A* is defective for twitching motility. (A) Stained subsurface twitching zones (left) and contrast imaging of surface colony edges (right) (the red arrow indicates the starting colony edge). (B) Average twitching zone measurements on the subsurface and surface. Download FIG S4, TIF file, 28.1 MB. Copyright © 2018 Little et al. 2018 Little et al. This content is distributed under the terms of the Creative Commons Attribution 4.0 International license.

In order to determine if the pyoverdine increase in strain PAO1 Δ*algR* could be complemented, PAO1 Δ*algR* containing an *algR*-complementing plasmid, pCMR-7, was compared to PAO1 and PAO1 Δ*algR* in the CAS assay. When supernatants from all three strains were compared for relative abilities to chelate iron, the PAO1 Δ*algR* supernatant chelated >1.5× more iron than that of the wild type, while *algR* complementation returned the pyoverdine production phenotype to wild-type levels (Fig. 1B). These results also indicated that the AlgR unphosphorylated form repressed pyoverdine synthesis and were consistent with the absorbance data (Fig. S3A).

To determine if AlgR-dependent pyoverdine control was applicable to other *P. aeruginosa* isolates, supernatants from *P. aeruginosa* strains PAK, mucoid PDO300, the mucoid CF clinical isolate FRD1, and their respective isogenic Δ*algR* strains, were examined for pyoverdine production. The pyoverdine levels were elevated in the Δ*algR* mutant strains of PAK, PDO300, and FRD-1 (Fig. S3B). Taken together, these data strongly indicate that AlgR represses pyoverdine production in *P. aeruginosa* mucoid, nonmucoid, laboratory, and clinical isolates.

Because previous data strongly indicated that AlgR D54N repressed pyoverdine production (Fig. 1B), we hypothesized that increased amounts of AlgR D54N would further decrease pyoverdine production. In order to test this hypothesis, the genes encoding AlgR D54N and AlgR D54E were placed into the arabinose-inducible plasmid pHERD30T and introduced into the PAO1 Δ*algR* background, and the iron-chelating capacity was assayed over increasing levels of AlgR induction. Increased AlgR D54E induction resulted in no change in chelation capacity until the highest level of induction (Fig. S3C). However, increased induction of AlgR D54N readily reduced the chelation capacity in a dose-dependent manner, down to 43% of the parental PAO1 Δ*algR* strain (Fig. S3C). These data strongly support the hypothesis that unphosphorylated AlgR repressed siderophore production and that AlgR phosphorylation relieves this repression.

### AlgR directly regulates *prrf2* transcription.

**FIG 2:**
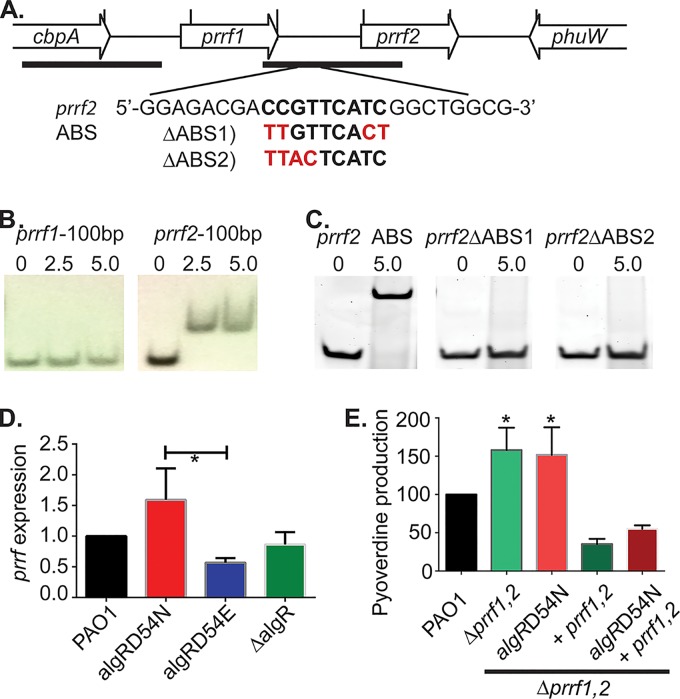
AlgR specifically regulates the promoter region of *prrf2*. (A) Schematic diagram of the *prrf1* and *prrf2* genomic region, with vertical bars denoting 100-bp increments, horizontal bars indicating PCR fragments used for the blots shown in panel B and the 25-bp region in panel C, including point mutations (in red). (B) EMSA results for potential AlgR binding sites upstream of *prrf1* and *prrf2*, using the indicated concentration (micromolar) of AlgR. (C) EMSA results for the 25-bp ABS region identified as the AlgR binding site upstream of *prrf2*, for which we used 5 µM AlgR, with the mutations ΔABS1 and ΔABS2 (diagrammed in panel A). (D) RT-qPCR of the *prrf1* and *prrf2* region in LB when we used the strains described for Fig. 1. (E) Double chromosomal mutants of both *prrf1* and *prrf2* (PAO1Δ*prrf1,2*) and PAO1*algR D54N*, with and without a complementing *prrf1,2* plasmid (pUCP19-*prrf1,2*), were compared for iron chelation of isolated supernatants in liquid CAS medium. Data were analyzed by ANOVA with a Bonferroni multiple-comparison test. Asterisks denote comparison to PAO1, except where noted by a bar. *, *P* < 0.05; **, *P* < 0.01; ***, *P* < 0.001.

The data detailed above showed that AlgR has a strong influence on pyoverdine production, and so we sought to determine the mechanism by which this was controlled. A previous study indicated that deletion of the *prrf1* and *prrf2* small regulatory RNAs resulted in increased production of siderophores (44), and the AlgR ChIP-seq data identified a single binding site between the noncoding RNAs *prrf1* and *prrf2* (32). The *prrf1* and *prrf2* small regulatory RNAs are functional analogues of the *rhyB* sRNA of *Escherichia coli*, which regulates iron homeostasis via the degradation of target mRNAs (45). The sequences of *prrf1* and *prrf2* are >95% identical, and data thus far do not indicate that deletion of one or the other has an independent phenotypic affect (45). A previous study demonstrated that deletion of both *prrf1* and *prrf2* sRNAs increased production of siderophores, indicating that one or both of these genes affect siderophore production either directly or indirectly (44). Here, the direct regulation of *prrf1* and *prrf2* expression through AlgR was investigated. The *prrf1* and *prrf2* promoter regions were examined via an electrophoretic mobility shift assay (EMSA) with both PCR products (Fig. 2A, horizontal bars, 100-bp promoter region centered around predicted AlgR binding sites) and hybridized 25-bp oligonucleotides containing a potential AlgR binding site (ABS) (Fig. 2A). In agreement with the previous ChIP-seq data, AlgR bound the *prrf2* promoter region but not the *prrf1* promoter region (Fig. 2B). In order to determine if the AlgR consensus binding sequence was required for AlgR binding, 25-bp fragments were generated, centering around the proposed AlgR binding site either containing the wild-type binding site or two different mutations of the 9-bp AlgR consensus binding sequence. Replacing four of the conserved bases in the 9-bp AlgR consensus binding site abrogated binding (Fig. 2C). To determine how *prrf* expression was being affected, RT-qPCR was performed in the *algR* mutant backgrounds (PAO1 *algR D54N*, PAO1 *algR D54E*, and PAO1 Δ*algR*). The RT-qPCR expression data indicated that unphosphorylated AlgR, encoded in PAO1 *algR D54N*, resulted in increased expression of the *prrf1* and *prrf2* genes relative to that of PAO1 *algR D54E*, which expressed the constitutively phosphorylated form of AlgR (Fig. 2D). The deletion strain PAO1 Δ*algR* did not, however, show any differential expression of *prrf1* and *prrf2*. Due to the high sequence identity between *prrf1* and *prrf2* (>95%), RT-qPCR cannot distinguish between these individual sRNAs, suggesting that the effect on *prrf2* may be larger but diluted to a certain degree by background *prrf1*.

Because increased *algR D54N* expression decreased pyoverdine expression (Fig. S3C) and increased *prrf* expression (Fig. 2D), we sought to determine if the changes in pyoverdine expression were due to AlgR D54N or *prrf*. In order to test this hypothesis, *algR D54N* was placed onto the strain PAO1 Δ*prrf1,2* chromosome to create PAO1 Δ*prrf1,2 algR D54N*. The *prrf1* and *prrf2* genes were complemented with a plasmid back into both strains, and pyoverdine production was measured. There was no difference in the pyoverdine produced by strains PAO1 Δ*prrf1,2* and PAO1 Δ*prrf1,2 algR D54N* (Fig. 2E). Introduction of *prrf1* and *prrf2* into either strain PAO1Δ *prrf1,2* or PAO1Δ *prrf1,2 algR D54N* reduced the iron-chelating capacity to below wild-type levels (Fig. 2E). These results suggested that strain PAO1 *algR D54N* decreased iron-chelating capacity through *prrf1* and *prrf2*. Because the expression plasmid for *prrf1* and *prrf2* drove the iron-chelating capacity below wild-type levels, there may also have been increased amounts of the *prrf1* and *prrf2* sRNAs from the complementing plasmid. However, strain PAO1 Δ*algR* produced more pyoverdine than wild-type PAO1, with no observable difference in *prrf* expression levels (Fig. 1B and 2D). These data indicate that strains PAO1 *algR D54N* and PAO1 *algR D54E* have different *prrf1* and *prrf2* expression levels that may account for the differences in siderophore production. In contrast, strain PAO1 Δ*algR* had increased siderophore production with no changes in *prrf1* and *prrf2* expression, indicating that additional alternative regulatory mechanisms may also need to be considered.

### AlgR directly regulates *pvdS* expression.

One of the alternative regulatory mechanisms could be through the AlgR-dependent expression of the sigma factor PvdS, as several different genes associated with pyoverdine production were differentially regulated when the different *P. aeruginosa* strains with altered *algR* alleles were compared (Table S2, part A). In fact, four pyoverdine genes were statistically differentially expressed when we compared strain PAO1 *algR D54E* to PAO1 *algR D54N* (*pvdF, pvdD*, *pvdI*, and *pvdX*; *P* = 0.02, 0.05, 0.04, 0.001, respectively, via analysis of variance [ANOVA]). These results indicated that AlgR influenced pyoverdine production either through multiple promoters or through a master regulator like *pvdS*. Because expression levels of *pvdF*, *pvdD*, and *pvdI* are PvdS dependent (46) and *pvdS* expression was increased 5.9-fold (*P* = 0.07; ANOVA) in strain PAO1 *algR D54E* compared to strain PAO1 *algR D54N* (Table S2), direct AlgR regulation of *pvdS* expression was investigated by using RT-qPCR, Western blotting, and EMSA.

**FIG 3:**
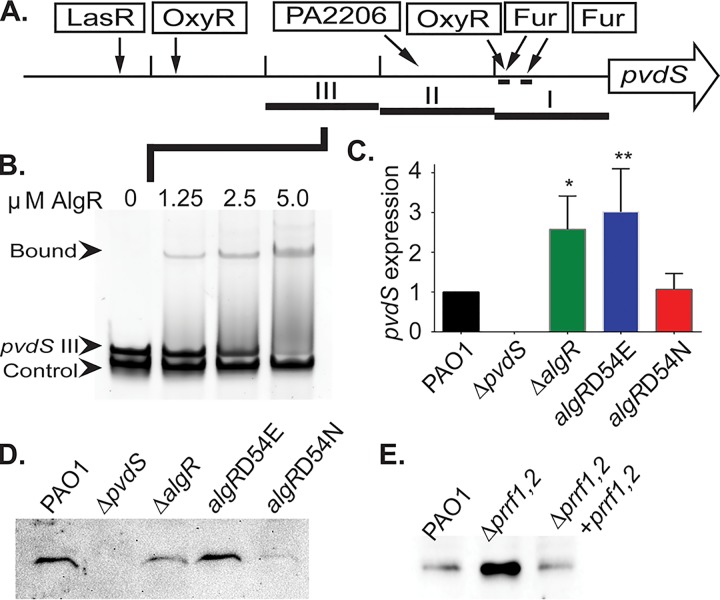
AlgR elicits a repressive effect on *pvdS* expression. (A) Schematic diagram of the *pvdS* promoter region, with vertical ticks denoting 100-bp increments, known protein binding sites, and noted 100-bp promoter fragments (I, II, and III). (B) EMSA results for the indicated 100-bp upstream fragment of *pvdS* (III) with an in-reaction negative binding control (*pscF*), using 1.25, 2.5, and 5 µM AlgR. Arrows on the left side of the gel indicate, from bottom to top, unbound *pscF*, unbound *pvdS*, and bound *pvdS*. (C) RT-qPCR of *pvdS* expression relative to the control gene *rpoD* using the strains defined in Fig. 1 (results were analyzed by ANOVA with a Bonferroni multiple-comparison test, compared to PAO1). *, *P* < 0.05; **, *P* < 0.01. Western blot analysis for PvdS expression with *algR* mutants (D) and *prrf1* and *prrf2* mutants (E) with a monoclonal anti-PvdS antibody.

In order to determine if AlgR bound the *pvdS* promoter, three 100-bp *pvdS* promoter fragments were evaluated by EMSA for AlgR binding (Fig. 3A, denoted as fragments I, II, and III). The first two 100-bp fragments (I and II) nearest to the gene showed only weak to no binding (data not shown), but the third fragment furthest (5′) from the translational start site was specifically bound by AlgR (Fig. 3B). As this binding was not competed when we used a nonspecific 95-bp *pscEF* promoter fragment in the reaction mixtures, this indicates that AlgR binding is specific for that fragment of the *pvdS* promoter region.

When RT-qPCR was used to evaluate expression from the *pvdS* promoter, both strains PAO1 Δ*algR* and PAO1 *algR D54E* showed elevated levels of *pvdS* expression compared to wild-type PAO1, whereas PAO1 *algR D54N pvdS* expression levels were not different from those of PAO1 (Fig. 3C). PvdS expression levels were also examined by Western blotting, using an anti-PvdS antibody. While PAO1 *algR D54E* had increased levels of PvdS, similar to RT-qPCR observations, strain PAO1 Δ*algR* did not show elevated PvdS levels by Western blotting (Fig. 3D). As the PAO1 Δ*algR* strain has been shown to increase pyoverdine production (Fig. 1B; Fig. S3), regulation in this background may involve other pyoverdine biosynthetic genes, or alternative posttranscriptional regulatory mechanisms of pyoverdine production. The other genes or additional regulatory mechanisms involved in pyoverdine regulation in the PAO1 Δ*algR* background are speculative at this point, but our transcriptional profiling did yield several poorly understood iron-associated regulatory proteins whose expression was altered by AlgR (Table S2, part A).

We observed an increased iron-chelating capacity in the PAO1 Δ*prrf1,2* and PAO1 Δ*prrf1,2 algR D54N* strains (Fig. 2E); therefore, Western blotting with anti-PvdS was performed to determine if *prrf1* and *prrf2* deletion and complementation affected PvdS expression. Our data showed that *prrf1* and *prrf2* deletion increased PvdS expression, which was complemented back to wild-type levels by introduction of the plasmid carrying *prrf1* and *prrf2* plasmid (Fig. 3E). These data indicate that AlgR D54N most likely activates *prrf2* which decreases PvdS expression and hence pyoverdine production. It should be mentioned that deletion of one or both *prrf* genes resulted in increased expression of genes involved in iron storage and utilization (e.g., *brfB* and tricarboxylic acid cycle enzymes) under iron-limiting conditions (45, 47).

### AlgR alters pyoverdine production when grown on different carbon sources.

Pyoverdine production increases as iron-limiting conditions are encountered by the organism (48–50), and decreasing iron concentrations stimulate alginate production (33). Therefore, the possibility that AlgR responds to changes in iron concentration was tested. Because the *fimU* promoter is AlgR dependent (31) and expression coincides with pyoverdine production (Fig. S1), strains PAO1 and PAO1 *algR D54E* containing a single-copy chromosomal *fimU*::*lacZ* fusion were grown on iron-depleted dialyzed tryptic soy broth (DTSB) medium both with and without the addition of 100 µM iron. No changes in *fimU* expression were observed with different iron concentrations (Fig. S5A). Because *fimU* expression is dependent upon AlgR phosphorylation, it may be surmised that there was no change in the AlgR phosphorylation state between the tested iron concentrations.

10.1128/mBio.02318-17.6 FIG S5  Iron concentration does not alter *fimU* activity, and the effect of AlgR does not overcome iron repression of siderophore production. (A) Measurement of *fimU*::*lacZ* activity from strains PAO1 and PAO1 *algR D54E* when grown in iron-deplete (black) or iron-replete (red) medium. (B) Production of pyoverdine by strains PAO1 and PAO1 *algR D54E* when grown with increasing iron concentrations (0 to 100 µM, black to darkening shades of red). Data were analyzed by ANOVA with a Bonferroni multiple-comparison test; bars denote comparisons. **, *P* < 0.01; ns, not significant. Download FIG S5, TIF file, 22.3 MB. Copyright © 2018 Little et al. 2018 Little et al. This content is distributed under the terms of the Creative Commons Attribution 4.0 International license.

Expression of *pvdS* and pyoverdine production are negatively regulated by increasing iron concentrations through Fur (48–50). Because pyoverdine production is regulated by Fur and AlgR, we sought to determine which regulator affected pyoverdine production over a range of iron concentrations. To examine this, strains PAO1 and PAO1 *algR D54E* were grown with increasing amounts of iron (0, 25, 50, 100 µM FeCl_3_), and pyoverdine production was measured (Fig. S5B). If AlgR-dependent pyoverdine production is responsive only to changing iron concentrations by phosphorylation, then strain PAO1 *algR D54E* should not respond and should be constitutively elevated. As observed previously, strain PAO1 *algR D54E* produced more siderophores than the wild type when grown in LB medium with no added iron. However, as increasing iron concentrations reduced pyoverdine production in PAO1, the same result was obtained from strain PAO1 *algR D54E* supernatants and resulted in a loss of statistical difference between strains PAO1 and PAO1 *algR D54E* (Fig. S5B). These data indicate that Fur, or another regulator besides AlgR, exerts a stronger influence over pyoverdine production as the cell encounters conditions more replete with iron. As AlgR does not directly respond to changes in iron concentration (Fig. S5A), it is not surprising that factors directly sensing iron (i.e., Fur) would override the AlgR influence on iron-associated gene expression.

**FIG 4:**
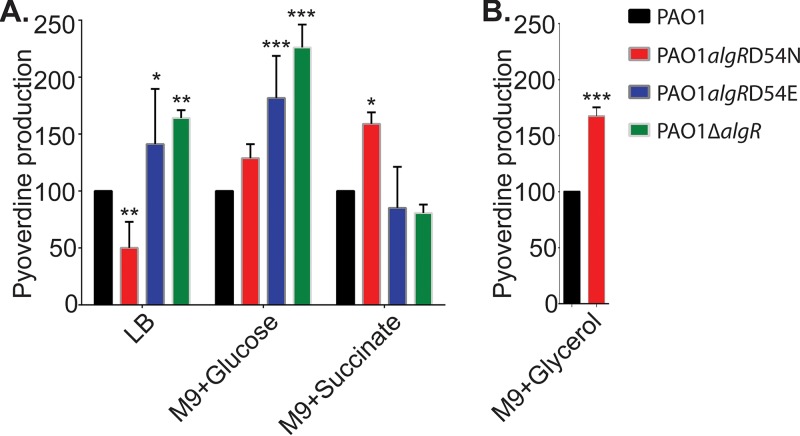
Effect of carbon sources on pyoverdine production between *algR* mutant strains. Production of pyoverdine by PAO1, PAO1 *algR D54N*, PAO1 *algR D54E*, and PAO1 Δ*algR* grown in LB or M9 minimal medium with either glucose, succinate, or glycerol (only PAO1 and PAO1 *algR D54N*) as the sole carbon source. Data were analyzed by ANOVA with a Bonferroni multiple-comparison test. Asterisks denote comparison to PAO1: *, *P* < 0.05; **, *P* < 0.01; ***, *P* < 0.001.

According to the AlgR-dependent gene expression levels revealed in our microarrays (Table S2, part D), the expression levels of numerous carbon utilization genes were affected by the AlgR phosphorylation state. Additionally, the AlgR ChIP-seq study revealed that 32% of the 157 identified binding sites were within or upstream of genes involved in carbon metabolism (32). Therefore, the hypothesis that AlgR-dependent pyoverdine production may be impacted by changes in carbon source was tested. Strains PAO1, PAO1 *algR D54N*, PAO1 *algR D54E*, and PAO1 Δ*algR* were grown in M9 minimal medium with glucose or succinate as the sole carbon source (Fig. 4). Compared to cells grown in Miller LB medium, there were surprising differences in pyoverdine production, but not growth, between the strains. Strains PAO1 *algR D54E* and PAO1 Δ*algR* continued to produce more pyovderine than the wild type when glucose was the sole carbon source, but when succinate was the sole carbon source, strain PAO1 *algR D54E* was indistinguishable from PAO1 and strain PAO1 Δ*algR* had a lower iron-chelating capacity than the wild type (Fig. 4A). Strain PAO1 *algR D54N* produced similar pyoverdine levels as the wild type in the minimal medium with glucose but more than the wild type when grown with succinate or glycerol, despite producing less pyoverdine in LB (Fig. 4B). Taken together, these data indicate that the AlgR phosphorylation state affects carbon utilization pathways that influence pyoverdine production.

### PAO1 and PAO1 *algR D54E* phenocopy virulence in a *Drosophila melanogaster* feeding infection model.

Since pyoverdine and pyocyanin production were inversely regulated by AlgR phosphorylation and abrogating their production affects virulence (13, 51–54), we determined whether AlgR phosphorylation affects virulence. *P. aeruginosa* strains PAO1, PAO1 *algR D54N*, PAO1 *algR D54E*, PAO1 Δ*algR*, PAO1 Δ*algZR*, and PAO1 *algZ H175A* were tested in a *Drosophila melanogaster* acute infection feeding model. Feeding and survival were followed for 14 days (Fig. S6). Flies infected with strain PAO1 or PAO1 *algR D54E* showed high levels of mortality, with 80% and 75% succumbing to the infection by day 14 (20% and 25% survival, respectively) (Fig. S6A). Strain PAO1 *algR D54E* was not statistically different from PAO1 in this virulence model (*P* = 0.44, log rank test). Strain PAO1 Δ*algR* displayed 43% survival at day 14 (*P* = 0.04, log rank test), which was increased relative to that of the parental PAO1 strain (Fig. S6A). These results corroborated previous data that showed decreased virulence with this strain in a mouse septicemia model (36). The strain mimicking a constitutively unphosphorylated state of AlgR, PAO1 *algR D54N*, was defective for virulence relative to the parental PAO1 strain, with 57% survival (*P* = 0.0002, log rank test), indicating that AlgR phosphorylation is critical for the full virulence of *P. aeruginosa* in a *Drosophila* feeding infection model (Fig. S6A).

10.1128/mBio.02318-17.7 FIG S6  Effect of AlgR phosphorylation on virulence in a *Drosophila melanogaster* feeding model of infection. Flies were fed a sucrose solution containing *P. aeruginosa algR* mutants (strains described in the Fig. 2 legend) (A) or strain PAO1 *algZ* mutants (strains described for Fig. 3) and tracked for survival over 14 days. *, *P* < 0.05; **, *P* < 0.01; ***, *P* < 0.001 (log rank test). Download FIG S6, TIF file, 18.7 MB. Copyright © 2018 Little et al. 2018 Little et al. This content is distributed under the terms of the Creative Commons Attribution 4.0 International license.

If the strain encoding an AlgR that is unable to be phosphorylated (PAO1 *algR D54N*) is attenuated for virulence, then a *P. aeruginosa* strain that does not contain a functional form of the putative AlgR histidine kinase, AlgZ, should also be attenuated in the same infection model. The PAO1 Δ*algZR* strain showed 51% survival at day 14 (*P* = 0.0009, log rank test). Flies were also infected with PAO1 *algZ H175A* and followed for 14 days. Flies infected with strain PAO1 *algZ H175A*, which carries an AlgZ with a mutation at the predicted site of autophosphorylation, showed 50% survival to day 14 (Fig. S6B) (P = 0.001, log rank test). This is in contrast to the flies that were infected with strain PAO1 or PAO1 *algR D54E*, among which only 20% and 25% of flies survived the full 14 days, respectively (Fig. S6A). These data indicate that AlgR phosphorylation by AlgZ contributes significantly to *P. aeruginosa* virulence in a *Drosophila melanogaster* infection model.

### The *P. aeruginosa algR D54N* and Δ*algR* strains are reduced in virulence in a mouse pneumonia model.

**FIG 5:**
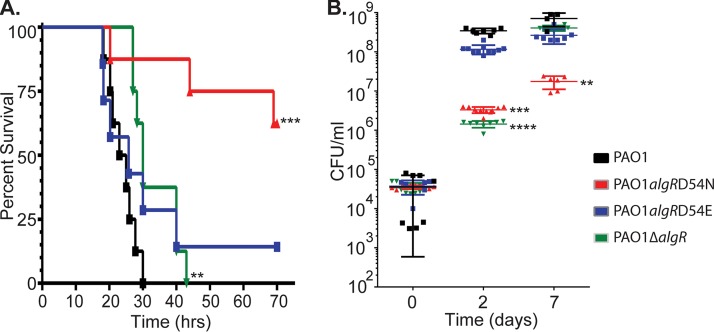
Effect of AlgR phosphorylation on virulence in an acute murine wound and pneumonia models of infection. (A) Mice intranasally inoculated with strains PAO1, PAO1 *algR D54N*, PAO1 *algR D54E*, and PAO1 Δ*algR* and followed over 72 h for survival. Results were analyzed with the log rank test. (B) Wounds infected with *algR* mutants (described for panel A) and followed over 7 days for infection establishment and maintenance (CFU counts per milliliter). Results were analyzed using the Kruskal-Wallis ANOVA test. *, *P* < 0.05; **, *P* < 0.01; ***, *P* < 0.001.

Murine acute pneumonia infection models are common in studying *P. aeruginosa*, as it is a well-known lung pathogen (55), and so we sought to use a pneumonia model to investigate whether AlgR phosphorylation affects virulence. Nine-week-old BALB/c mice were intranasally inoculated with 4 × 10^7^ CFU of either strain PAO1, PAO1 Δ*algR*, PAO1 *algR D54N*, or PAO1 *algR D54E*. Two replicate experiments were performed (4 mice per strain, 8 mice total per strain), and mouse survival was followed over a 72-h time period (Fig. 5A). Mice infected with strain PAO1 Δ*algR* displayed a longer mean time to death than those infected with PAO1 (*P* = 0.0289, log rank test). This result corroborated previous data showing decreased virulence of strain PAO1 Δ*algR* in a mouse septicemia model and more recent studies showing this same strain was attenuated in a similar infection model (32, 36). Mice infected with strain PAO1 *algR D54N* had a mean time to death that was significantly different from that after PAO1 infection (*P* = 0.0067). Mice infected with strain PAO1 *algR D54N* had 67% survival, compared to a 0% survival rate of the mice infected with PAO1 over the same time period. Mice infected with strain PAO1 *algR D54E* had 25% survival. Two mice infected with the strain PAO1 *algR D54E* were able to clear and survive infection. However, this result was not statistically significant compared to PAO1 (*P* = 0.1679), indicating that strain PAO1 *algR D54E* was as virulent as the wild type. In summary, PAO1 *algR D54N* was highly attenuated for virulence compared to the other three strains, indicating that AlgR phosphorylation is required for full virulence in the mouse pneumonia model.

### *P. aeruginosa* expressing *algR D54N* is attenuated in a murine acute wound infection model.

*P. aeruginosa* is also a common cause of surgical infections, and the role of AlgZR in wound infections has been suggested, as *P. aeruginosa* infection of rat wounds causes upregulation of *algR* expression (37, 56). Therefore, *P. aeruginosa* virulence was examined in an acute full thickness punch wound murine model of infection (57, 58). C57BL/6J mice were infected with PAO1, and the CFU burdens of the wounds were determined over 14 days (Fig. S7). The initial inoculum of 3 × 10^6^ CFU PAO1 was elevated to 3.5 × 10^8^ CFU by day 2. This level of bacterial burden was maintained for at least 8 days. As this infection model was not lethal in mice, the bacteria were eventually cleared from the wounds. This was evident at day 14, when the median wound CFU dropped to almost the initial inoculum at 4 × 10^6^ CFU, with some wounds containing as low as 6.5 × 10^2^ CFU (Fig. S7).

10.1128/mBio.02318-17.8 FIG S7  Kinetics of murine wounds infected with strain PAO1. (A) Acute wounds inoculated with PAO1; CFU (per milliliter) were quantified over 14 days of infection. Download FIG S7, TIF file, 10.2 MB. Copyright © 2018 Little et al. 2018 Little et al. This content is distributed under the terms of the Creative Commons Attribution 4.0 International license.

After establishing the PAO1 murine wound course of infection, we determined the impact that *algR* mutations have in this infection model. We examined infected wounds for CFU burden of PAO1, PAO1 Δ*algR*, PAO1 *algR D54E*, and PAO1 *algR D54N* (Fig. 5B). C57BL/6J wounds were inoculated with 4 × 10^4^ CFU and examined at 2 and 7 days postinoculation. At 2 days postinoculation, both PAO1 and PAO1 *algR D54E* thrived in the wounds, reaching average CFU of 3.4 × 10^8^ and 1.2 × 10^8^, respectively. Both PAO1 Δ*algR* and PAO1 *algR D54N* lagged behind on growth within the wound, with average CFU of 1.4 × 10^6^ and 3.3 × 10^6^ at day 2, respectively. At 7 days postinfection, both strains PAO1 and PAO1 *algR D54E* remained at increased levels, with averages of 7.1 × 10^8^ and 2.6 × 10^8^ CFU, respectively. PAO1 Δ*algR* recovered to an average CFU of 4.0 × 10^8^ at this time point. PAO1 *algR D54N* remained attenuated, with an average CFU of 1.7 × 10^7^ (Fig. 5B). Taken together, these results indicate that AlgR phosphorylation is critical for the organism to establish virulence in an acute murine wound model.

## DISCUSSION

Our data showed that: (i) AlgR phosphorylation was maximal in stationary phase; (ii) it coordinated pyocyanin and pyoverdine gene expression and; (iii) it directly affected virulence. There are conflicting data in the literature regarding the role of AlgR phosphorylation and gene expression, depending upon the promoter examined. The phosphomimetic AlgR (AlgR D54E) activated *fimU* and *rhlA* expression, while the phosphoablative AlgR (AlgR D54N) did not express *fimU* and decreased *rhlA* expression (27, 31). These data indicated that phosphorylation was required for *fimU* and modulated *rhlA* expression. In contrast, AlgR phosphorylation was not required to activate *algD* expression (59). Together, these data suggest that AlgR can activate gene expression regardless of phosphorylation state. Our data here suggest that the AlgR phosphorylation state coordinates different iron acquisition systems in response to growth phase and *in vivo* conditions. Because AlgR likely exists in a phosphorylation flux as a response to stimuli, we utilized mutated forms of AlgR that mimicked phosphorylation states to determine the importance of AlgR phosphorylation in gene expression and virulence.

Previous work had shown that AlgR was most highly expressed during stationary phase, indicating that this growth phase is when AlgR is most important and potentially when AlgZ senses its signal (34). Since *fimU* expression is strictly AlgR phosphorylation dependent, we utilized *fimU* expression in PAO1 to determine when AlgR is most likely phosphorylated. Because *fimU* expression was maximal at 16 h, we utilized this time point to examine the transcriptional profiles of PAO1 expressing the phosphomimetic mutant AlgR D54E and the phosphoablative mutant AlgR D54N. Surprisingly, these profiles revealed three things: (i) except for 5 genes, the genes controlled by strains PAO1 and PAO1 *algR* D54E were identical; (ii) only 8 genes were common between our transcriptional profiling of PAO1 *algR* D54E versus PAO1 *algR* D54N and results of an AlgR ChIP-seq study (Table S2, part E) (32); (iii) 25 genes involved in iron metabolism or acquisition were differentially expressed. The first revelation strongly indicated that AlgR is phosphorylated in late stationary phase and was consistent with *fimU* expression levels being maximal at the same time point. Further examination showed that AlgR directly and indirectly controlled the expression of pyoverdine and pyocyanin genes through *pvdS*, *prrf1* and *prrf2*, and *czcR*.

The *pvdS* gene directly controls iron acquisition in *P. aeruginosa*, as it encodes an alternative sigma factor that drives pyoverdine biosynthetic gene expression as well as the expression of other genes (60). Expression of *pvdS* is Fur regulated, with two binding sites overlapping the −10 and −35 regions (60, 61). Additionally, at least three other regulators control *pvdS* expression, including LasR (62), OxyR (63), and PA2206 (64). All of these regulators bind to the *pvdS* promoter 200 bp 5′ to the translational start. The location of the AlgR binding site relative to all these factors does not imply any sort of direct interaction, therefore indicating that no competitive binding between these regulators occurs on the *pvdS* promoter. However, the fact that there is no direct overlap with any other known factor does not rule out that AlgR and any of these other regulators may work together to express *pvdS*. The LasR control of *pvdS* expression suggests the possibility that AlgR and LasR may coordinate *pvdS* expression. This is not the first time AlgR has been associated with quorum sensing, as AlgR directly controls *rhlA* expression in a phosphorylation- and biofilm-dependent manner (30, 31). Both OxyR and PA2206 are oxidative stress-responsive factors in *P. aeruginosa*, and while *algR* deletion impacts the organism’s ability to survive oxidative stress conditions, the exact mechanisms are unknown (36). Our data show that phosphorylated AlgR (encoded in strain *algR D54E*) can directly activate both *pvdS* and PvdS expression (Fig. 3C and D).

AlgR also indirectly controls pyoverdine production by controlling *prrf2* expression; this finding is consistent with a previous report showing that *prrf1* and *prrf2* coordinate iron homeostasis (44). The small RNAs *prrf1* and *prrf2* bind to and result in the degradation of target mRNAs, ultimately resulting in changes in iron homeostasis (45) under iron-limiting conditions. While microarray analysis of PAO1 Δ*prrf1,2* did not show siderophore genes that were dysregulated (45), changes in siderophore production were observed in the PAO1 Δ*prrf1,2* strain (65). Expression of *prrf1* and *prrf2* was previously only known to be directly regulated by Fur (45). Under iron-replete conditions, Fur binds close to the start of *prrf1* and *prrf2*, overlapping the −10 and −35 regions, and the spacing between *prrf1* and *prrf2* is 95 bp. The AlgR binding site is separated from the Fur binding site by 36 bp and is located 72 to 63 bp 5′ from the start of *prrf2*. DNase I footprinting assays and *prrf2* expression studies under different iron concentrations with Fur and AlgR would determine if these proteins interact on the *prrf2* promoter. While the reason for this differential regulation between *prrf1* and *prrf2* is unknown, it is likely that *prrf1* and *prrf2* are capable of binding slightly different target sequences, and AlgR is tied to regulating a gene or genes that may be targeted by *prrf2* but not *prrf1*. Alternatively, the binding of AlgR may somehow disrupt expression of a longer heme-associated sRNA called *prrH*, which results from transcriptional readthrough of *prrf1* and *prrf2* (66). Further study is necessary to dissect these possible scenarios.

**FIG 6:**
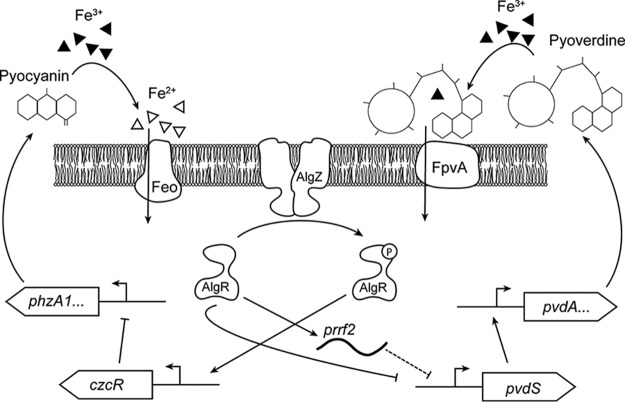
Model of AlgR’s role in pyoverdine and pyocyanin regulation. Unphosphorylated AlgR directly activates *prrf2* and represses *pvdS*, resulting in decreased pyoverdine production. Unphosphorylated AlgR does not interact with the *czcR* promoter (which encodes a negative regulator of the *phzA1* promoter), leading to increased pyocyanin. Phosphorylated AlgR no longer activates *prrf2* or represses *pvdS*, but it activates expression of *czcR*, leading to the repression of pyocyanin and increased pyoverdine.

Unlike the AlgR direct and indirect pyoverdine expression control, AlgR pyocyanin regulation appears to be indirect through *czcR*. *P. aeruginosa* pyocyanin biosynthesis involves two homologous core operons, *phzA1B1C1D1E1F1G1* and *phzA2B2C2D2E2F2G2* (67). CzcR is a reported pyocyanin repressor through direct binding of the *phzA1*, but not the *phzA2*, promoter (40). The *czcR* promoter region was discovered to be AlgR dependent based on ChIP-seq, *in vitro* EMSA, RT-qPCR, and pyocyanin assays (32). These previous data showed that *algR* deletion increased pyocyanin production, and increased *czcR* expression alone caused decreased pyocyanin production in the mutant Δ*algR* background to wild-type levels (32). However, the AlgR phosphorylation state was not examined. Our data show that both *algR* deletion and the phosphoablative form (AlgR D54N) increased pyocyanin production, consistent with the findings of Kong et al., and predict that *czcR* expression should be decreased in these strains. In contrast, phosphorylated AlgR decreased pyocyanin production, likely through increased *czcR* expression that would repress *phzA1* (Fig. 6). There is a conundrum between AlgR phosphorylation, which occurred in stationary phase (Fig. 1, Fig. S1), which should repress pyocyanin production, yet maximal pyocyanin expression levels have been observed in stationary phase (68). Pyocyanin production is under the direct control of the Rhl quorum-sensing system and is responsible for its increased expression levels in stationary phase (69, 70). Additionally, while CzcR has been described as a repressor of pyocyanin production through the *phz1* operon, we have observed changes in *phz2* operon expression in a PAO1 Δ*algR* strain grown in a biofilm (30). The role of CzcR in pyocyanin repression also needs further clarification, as *czcR* deletion increased pyocyanin and heterologous *czcR* expression complemented pyocyanin production. However, CzcR bound to the *phzA1* promoter but did not bind the *phzA2* promoter (40). This suggests that phosphorylated AlgR (AlgR D54E in our experiments) likely represses an additional activator or activating another repressor involved in pyocyanin production, as well as stimulating *czcR* expression. Alternately, one of the quorum-sensing system regulators overrides AlgR in phenazine gene expression.

While we identified three AlgR-dependent genes controlling iron acquisition, the microarray data suggest that there are still several other iron-associated genes that are under direct AlgR control (e.g., PA1300 and PA1301, PA2384, *hasS-hasI*). Interestingly, 13 of the 25 iron-related genes differentially regulated between strains *algR D54E* and *algR D54N*, are controlled by Fur (Table S2, part A). The ferric uptake regulator (Fur) binds promoters and represses expression under iron-replete conditions (71). Iron-limiting conditions result in the derepression of iron acquisition genes, including *pchR* and *pvdS*, which encode a transcriptional regulator that controls pyochelin production and the alternative sigma factor PvdS, respectively (60, 71–76). Although Fur has been extensively shown to regulate iron-associated genes, it is not the only iron acquisition regulatory factor. There is additional overlap between the RsmA-*rsmY/Z* and AlgZR regulatory systems with pyoverdine production (77). Conflicting data suggest that AlgZR may play a role in expression of *rsmA* and *rsmY/Z* (78–80), which may be a result of strain differences, e.g., AlgR’s effect on mucoid versus nonmucoid strains. While RsmA-*rsmY/Z* and *prrf1* and *prrf2* have been shown to influence production of siderophores, additional RNA mechanisms control other aspects of iron acquisition, including the readthrough of *prrf1* and *prrf2*, which produces *prrH*, a heme regulatory mechanism. Fur also directly regulates the heme uptake systems Has and Phu (81). The expression of *algZR* itself is also directly linked to heme biosynthesis, as *algR* is cotranscribed with the heme biosynthetic genes *hemC* and *hemD* and disruption of the *algZR* genes reduces *hemC* expression (34, 35). These observations suggest a relationship between the AlgZR system and various points in iron acquisition, both regulatory and through transcription of *algZR* itself. Taken together, it is tempting to speculate that one of the major roles for AlgZR in *P. aeruginosa* may be related to utilization of different iron sources for heme biosynthesis.

Iron acquisition systems are well known for their association with virulence in *P. aeruginosa*. *D. melanogaster* has been extensively used as a model of *P. aeruginosa* virulence with multiple infection systems (82, 83). Previous work has also identified the importance of RhlR in controlling the *Drosophila* immune response during infection (84), as well as production of cyanide (85), both of which are systems known to be regulated by AlgZR (29, 30).

The role of AlgZR in wound infections has been previously suggested, as *P. aeruginosa*-infected rat wounds showed increased *algR* expression (37). This model, along with the more common *P. aeruginosa* infection model, the murine pneumonia model, both indicated that the phosphoablative strain PAO1 *algR D54N* was consistently attenuated. There have been several previous virulence studies that examined the role of AlgR in either septicemia or pneumonia models (32, 36). These studies did not, however, examine the use of locked phosphorylation states but rather analyzed whole-gene deletions, which presents an issue because AlgR is expressed throughout all growth phases (34). The only condition in which a Δ*algR* strain was more virulent than the wild type was in neutropenic mice, indicating that a component of the immune system contributes to the lack of virulence in the other models (36). This likely also plays into the differences with the phosphorylated forms as well, as histology of both lungs and wounds showed considerably less inflammation and immune invasion with the PAO1 *algR D54N* strain (data not shown). The reason for this difference in immune system involvement is, however, unknown, and further research may yield interesting insights into the role of AlgR in regulating factors which interact with the immune system. The important point from these studies is that AlgZR plays an important role in virulence of the organism, and alterations of this system vastly impact its virulence.

One of the most surprising results from this study was AlgR phosphorylation control of iron acquisition through different carbon sources. While it was not particularly surprising that the role of AlgR in pyoverdine production could be overcome by the addition of iron, changes in pyoverdine production in response to different carbon sources was unexpected. There has been an indication from previous data that this was possible in *Pseudomonas pudita*, in which metabolic changes in carbon utilization under low-iron conditions changed siderophore expression levels (86). Our data suggest that AlgZR may direct a similar process in *P. aeruginosa*. Another indication that AlgZR controls carbon flux in *P. aeruginosa* is from the Chip-seq study, in which 32% of the AlgR-bound DNA fragments sequenced were associated with carbon metabolism genes (32). This information, along with the ability to manipulate pyoverdine production with different carbon sources, indicates that carbon metabolism may be a central component of the AlgZR system.

## MATERIALS AND METHODS

### Bacterial strains, plasmids, genetic manipulations, and growth conditions.

Bacterial strains, plasmids, and oligonucleotides utilized in this study are listed in Table S1. *P. aeruginosa* growth was at 37°C in LB-Miller or *Pseudomonas* isolation agar (PIA) (Difco).

10.1128/mBio.02318-17.9 TABLE S1  Strains, plasmids, and oligonucleotides used in this study. Download TABLE S1, DOCX file, 0.2 MB. Copyright © 2018 Little et al. 2018 Little et al. This content is distributed under the terms of the Creative Commons Attribution 4.0 International license.

10.1128/mBio.02318-17.10 TABLE S2  Microarray gene lists (the gene lists were generated from comparisons of strains PAO1, PAO1 *algR D54E*, and PAO1 *algR D54N*, as described in Materials and Methods). (A) AlgR-dependent iron genes from the three strains. (B) Strain PAO1 versus PAO1 *algR D54N* with genes that were significantly differentially expressed (ANOVA, *P* < 0.05) more or less than 2-fold compared to PAO1. (C) Strain PAO1 versus PAO1 *algR D54E* genes that were statistically significantly (ANOVA, *P* < 0.05) expressed more or less than 2-fold compared to PAO1. (D) PAO1 *algR D54E* versus PAO1 *algR D54N* genes that were statistically significantly (ANOVA, *P* < 0.05) expressed more or less than 2-fold compared to PAO1 *algR D54E*. (E) AlgR expression versus ChIP-seq results for genes common to those reported by Kong et al. ChIp-seq (32) and the genes listed in part D of this table. Download TABLE S2, XLSX file, 0.1 MB. Copyright © 2018 Little et al. 2018 Little et al. This content is distributed under the terms of the Creative Commons Attribution 4.0 International license.

### RNA isolation and preparation for Affymetrix GeneChip analysis.

Total RNA samples were prepared from three independent replicates of *P. aeruginosa* strains PAO1, PAO1 *algR D54E*, and PAO1 *algR D54N* grown for 16 h in LB as previously described elsewhere (30).

### Pyocyanin and pyoverdine production assays.

Pyocyanin production was measured as previously described (87, 88). Detection of pyoverdine was performed by using a modified version of the CAS assay as described previously (43, 89).

### EMSA.

EMSA reactions were performed as described previously (31), utilizing either PCR-generated 100-bp fragments or hybridized 25-bp oligonucleotides.

### Additional information.

Additional details regarding our experimental procedures and materials are provided in Text S1 in the supplemental material.

10.1128/mBio.02318-17.1 TEXT S1  Supplemental materials and methods. Download TEXT S1, DOCX file, 0.1 MB. Copyright © 2018 Little et al. 2018 Little et al. This content is distributed under the terms of the Creative Commons Attribution 4.0 International license.
