# Supplementary figures and images for: Pseudomonas aeruginosa AlgR Phosphorylation Status Differentially Regulates Pyocyanin and Pyoverdine Production
Source: mBio. 2018 Jan 30;9(1):e02318-17. doi: 10.1128/mBio.02318-17 (PMC5790918; doi:10.1128/mBio.02318-17)

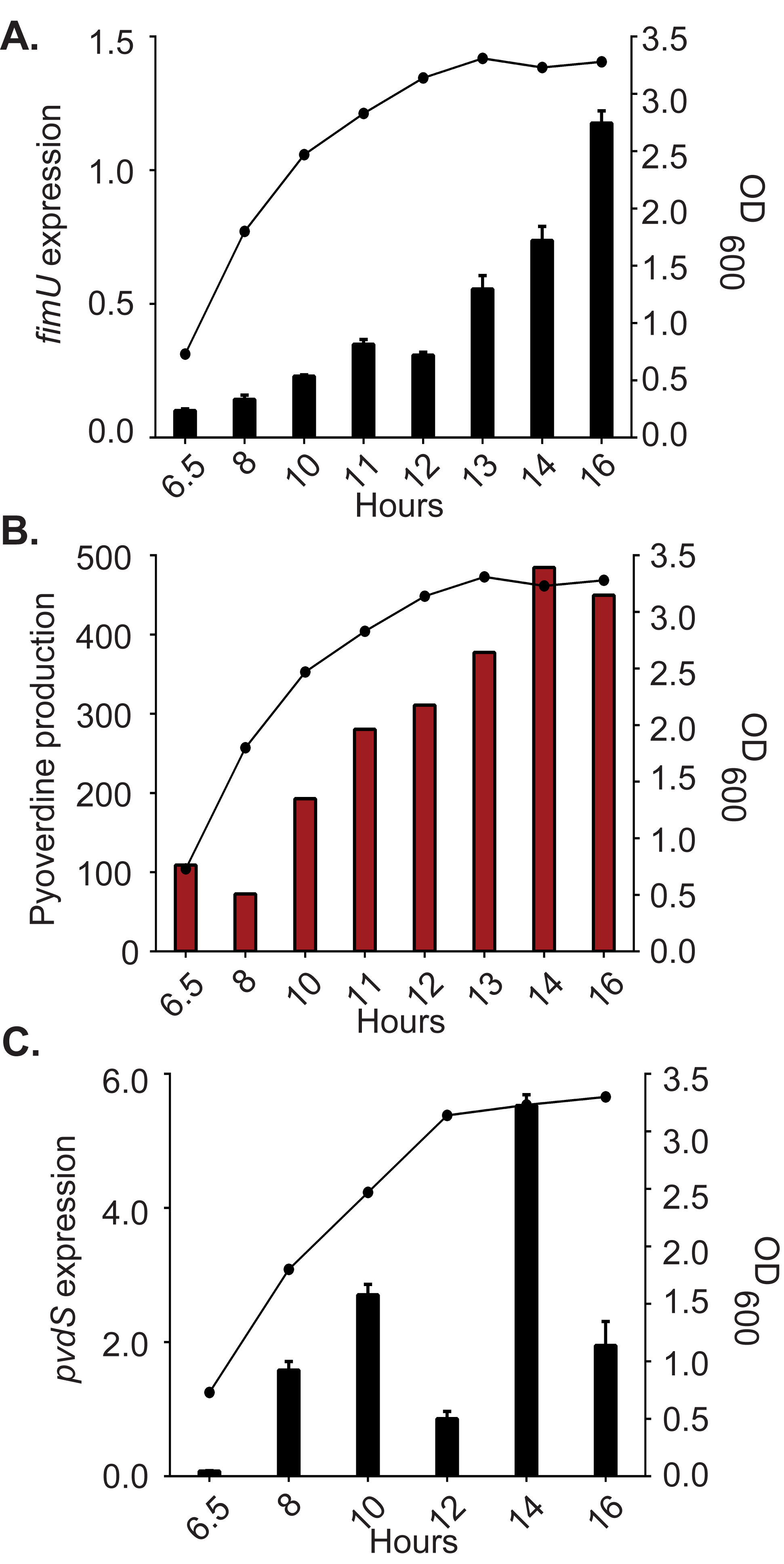

Supplement: FIG S1 [file mbo001183691sf1.tif]

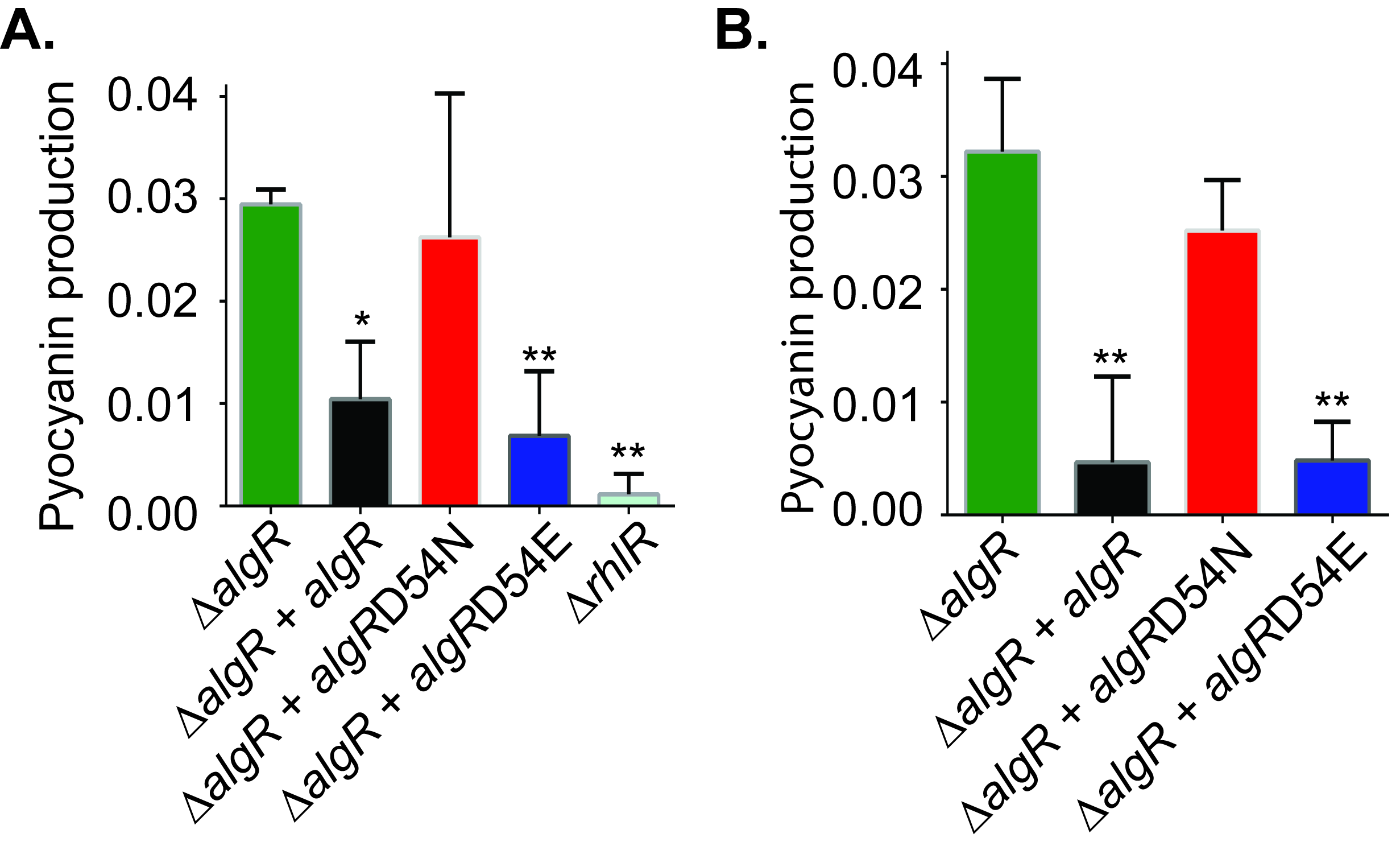

Supplement: FIG S2 [file mbo001183691sf2.tif]

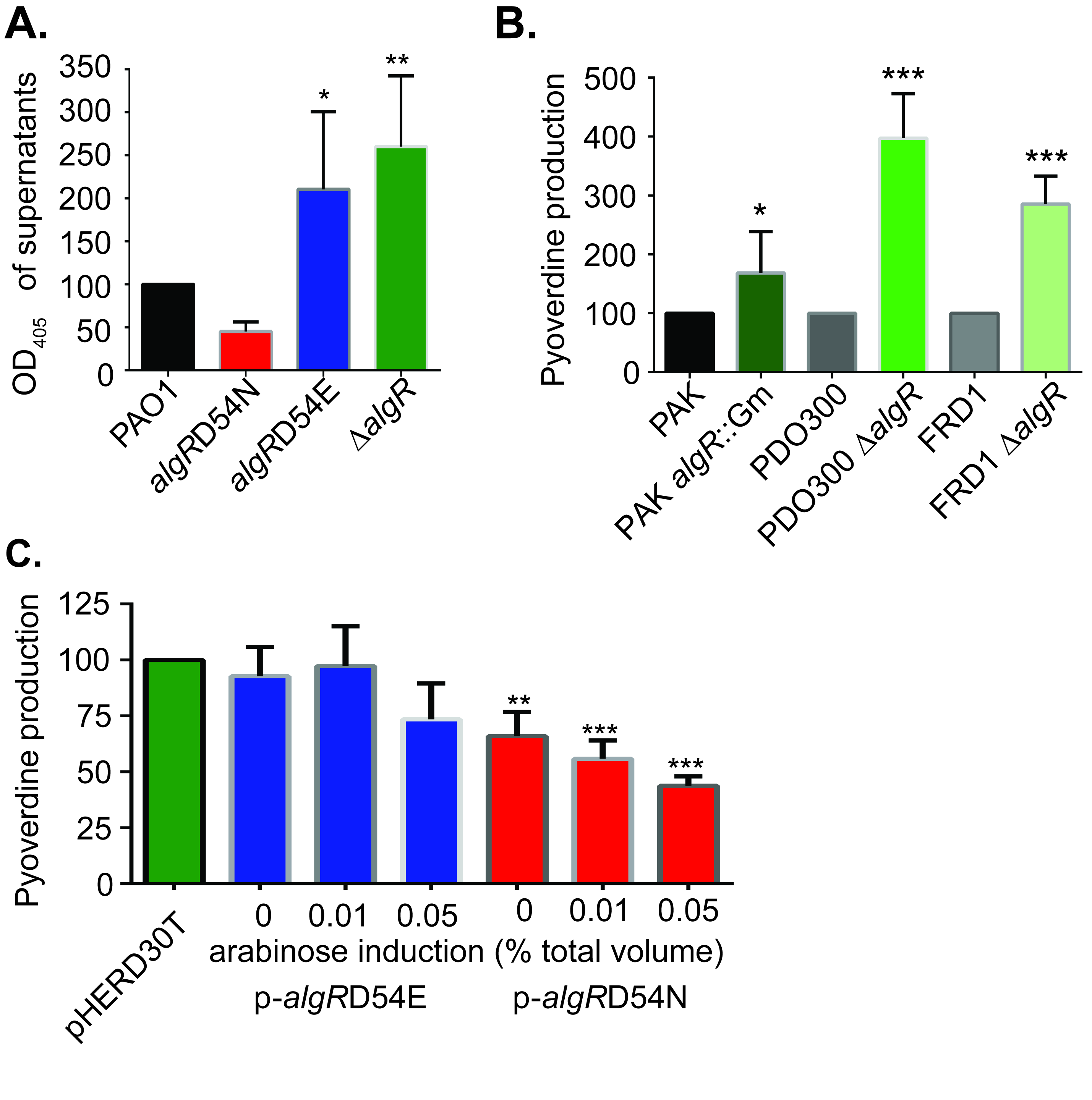

Supplement: FIG S3 [file mbo001183691sf3.tif]

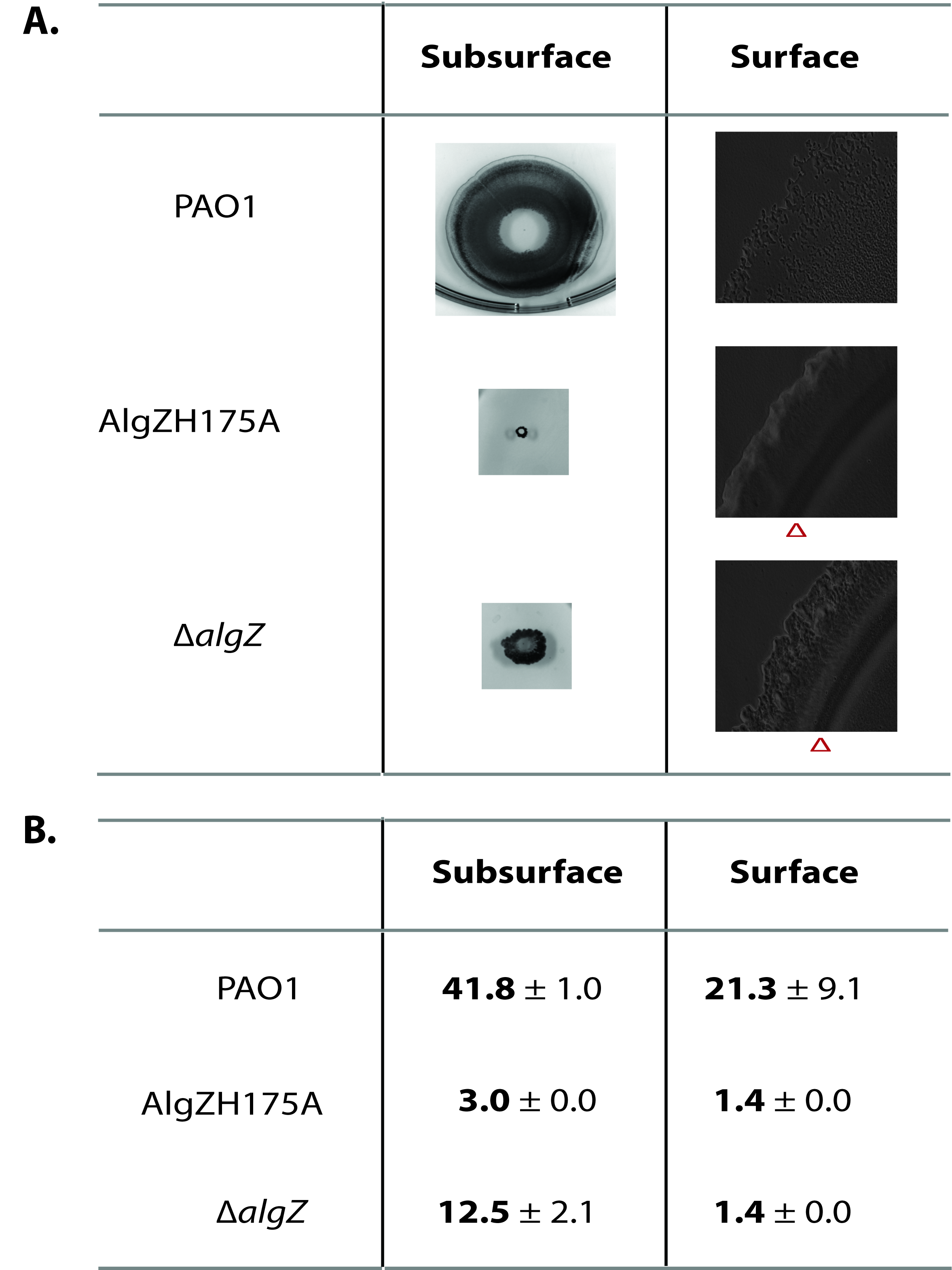

Supplement: FIG S4 [file mbo001183691sf4.tif]

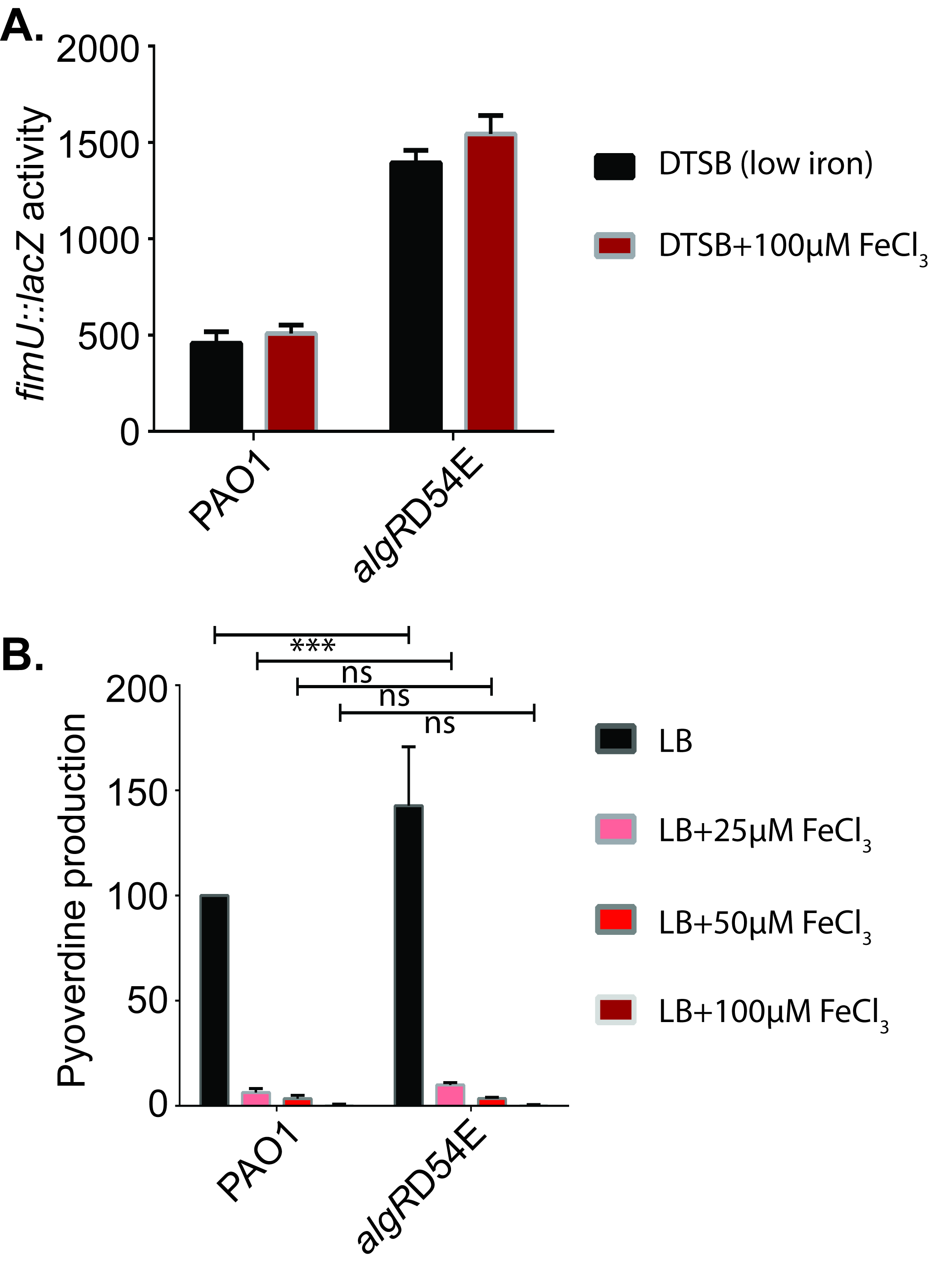

Supplement: FIG S5 [file mbo001183691sf5.tif]

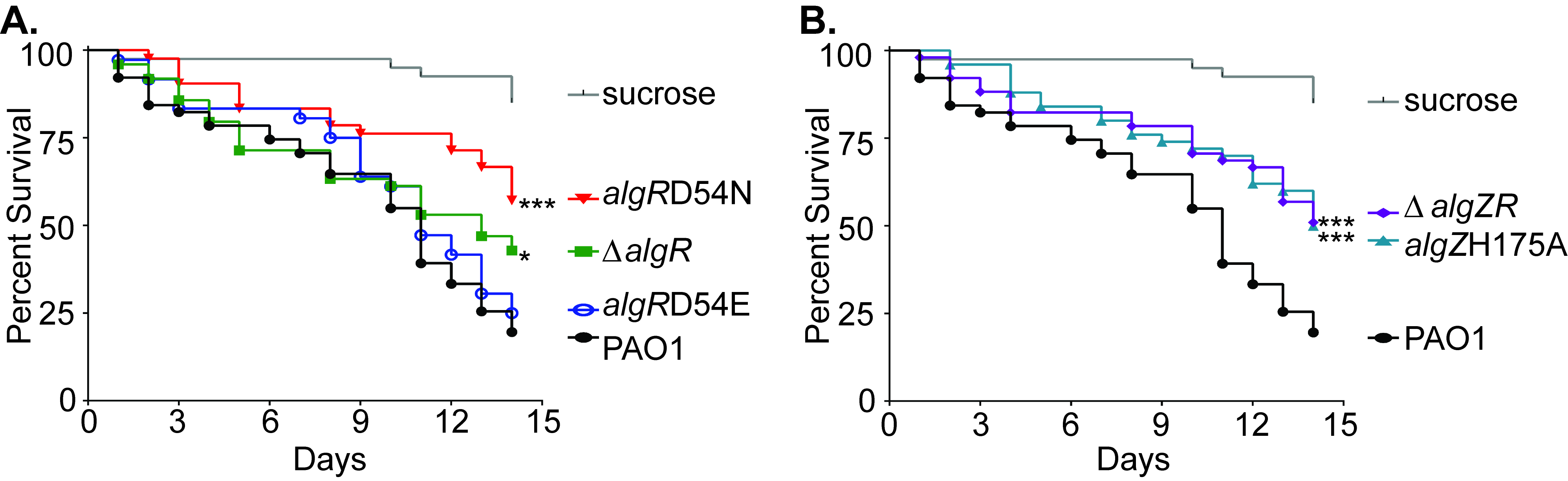

Supplement: FIG S6 [file mbo001183691sf6.tif]

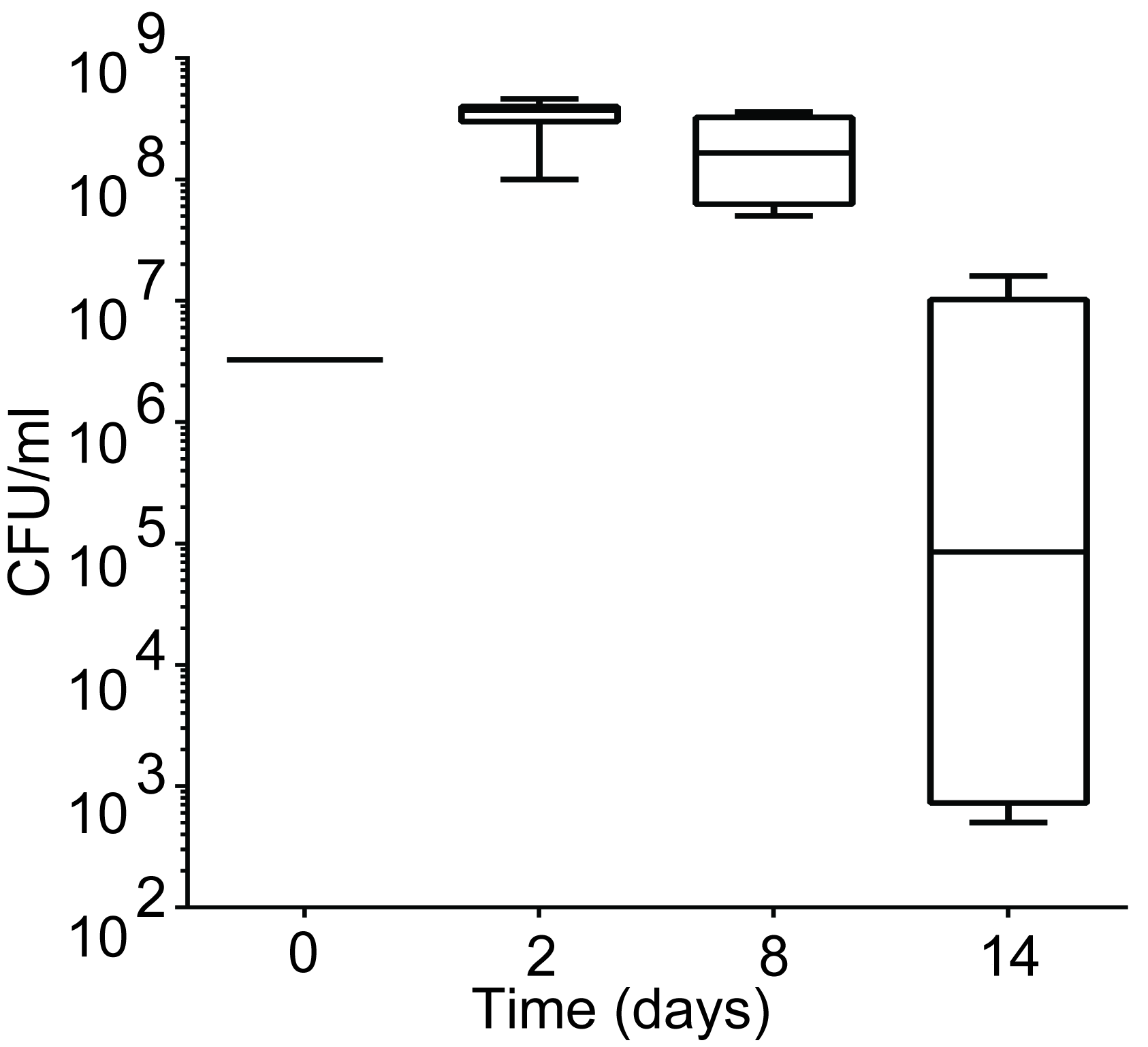

Supplement: FIG S7 [file mbo001183691sf7.tif]
