# Supplementary material for: Pseudomonas aeruginosa AlgR Phosphorylation Status Differentially Regulates Pyocyanin and Pyoverdine Production
Source: mBio. 2018 Jan 30;9(1):e02318-17. doi: 10.1128/mBio.02318-17 (PMC5790918; doi:10.1128/mBio.02318-17)
Supplement: TABLE S1 [file mbo001183691st1.docx]

Table S1. Strains, plasmid and oligonucleotides used in this study.

| Strain or plasmid | Relevant genotype or other characteristics | Reference or source |
| --- | --- | --- |
| *P. aeruginosa* |  |  |
| PAO1 | Wild type *P. aeruginosa* | (1) |
| PAK | Hyper piliated wild type *P. aeruginosa* | Matt Wolfgang |
| PSL317 | PAO1 Δ*algR* | (2) |
| PAK *algR::Gm* | *algR::Gm* | (3) |
| PAO1 *algR*D54E | *algR*D54E, encoding AlgR D54E | (4) |
| PAO1 *algR*D54N | *algR*D54N encoding AlgR D54N | (5) |
| PAZ | PAO1 Δ*algZ* | (6) |
| PAO1 *algZ*H175A | *algZ* H175A | This work |
| PAO1 Δ*algR*Δ*algZ* | Δ*algR* Δ*algZ* | This work |
| PAO1 Δ*pvdS* | Δ*pvdS* | Mike Vasil |
| PAO1 Δ*pvdA* | Δ*pvdA* | Mike Vasil |
| PAO1 Δ*pvdA*Δ*pchEF* | Δ*pvdA*Δ*pchEF* | Mike Vasil |
| PAO1 Δ*prrf1,2* | Δ*prrf1,2* | (7) |
| PDO300 | PAO1 *mucA22* | (8) |
| PDR300 | PAO1 *mucA22*; Δ*algR* | (9) |
| FRD-1 | Mucoid clinical isolate | Dan Hassett |
| FRD-1R | Mucoid clinical isolate; Δ*algR* | (10) |
| PAO1 *fimU::lacZ* | *fimU::lacZ* (in *attB* site) | (4) |
| PAO1 algRD54E *fimU::lacZ* | *fimU::lacZ* (in *attB* site) | (11) |
|  |  |  |
| *E. coli* |  |  |
| DH5α | F^−^ ϕ80dlacZΔM15 Δ(lacZYA-argF)U169 deoR recA1 endA1 hsdR17(r_K_^−^ m_K_^+^) phoA supE44 λ^−^ thi-1 gyrA96 relA1 | Lab strain |
|  |  |  |
| Plasmids |  |  |
| pHERD30T | pBAD-based *Pseudomonas* expression vector; Gm^r^ | (12) |
| pRK2013 | Tra^+^ Mob^+^ ColE1 replicon; Km^r^ | (13) |
| pFLP-2 | Source of FLP recombinase; Amp^r^ | (14) |
| pEXGMΔ*algZR* | *algZR*  deletion vector | (15) |
| pEXG2-*algZ*H175A | *algZ*H175A allelic exchange vector | This study |
| pHERD30T-AlgRD54E | algRD54E in pHERD30T; Gm^r^ | (11) |
| pHERD30T-AlgRD54N | algRD54N in pHERD30T; Gm^r^ | (11) |
| pUCP18-*prrf12* | pUCP18 shuttle vector containing *prrf12*; Cb^r^ | (16) |
| pCMR7 | pVD*tac*24 *algR* | (17) |

| Primer Name | 5’-3’ Sequence | Gene |
| --- | --- | --- |
| algZ-1212F XbaI | CCCTCTAGATGTCTTCCTGGTTGTCCTTGTTGTA | *algZ* |
| algZ+1077R HindIII | CCCAAGCTTTCAGGCTTCCTGCATGAGTC | *algZ* |
| algZH175AF-HpyAV | GCGCGGATTCGCCCGGCCTT | *algZ* |
| algZH175AR-HpyAV | AGGCTGTTGAACAGGAAGGCCGGG | *algZ* |
| rpoD-F-qPCR | CCTGATGAAGGCGGTGGAC | *rpoD* |
| rpoD-R-qPCR | GATGCGGATGGTGCGTGC | *rpoD* |
| fimU-F-qPCR | GCCGCCGAGGAACTCAATG | *fimU* |
| fimU-R-qPCR | GCCAGGACACCTATGCTCAGG | *fimU* |
| PrrHF | CCAGAAAGTCGGCGATGTGC | *prrf1* |
| PrrHRev | TCAGGCTATGGGCGATTCG | *prrf1* |
| Prrf2For | GGTCTTTTTTTGCCTGCGATTC | *prrf2* |
| Prrf2Rev | CCGACTGCTTGGTCTCTCAGC | *prrf2* |
| prrf2-neg1-F | GGAGACGATTGTTCACTGGCTGGCG | *prrf2* |
| prrf2-neg1-R | CGCCAGCCAGTGAACAATCGTCTCC | *prrf2* |
| prrf2-neg2-F | GGAGACGATTACTCATCGGCTGGCG | *prrf2* |
| prrf2-neg2-R | CGCCAGCCGATGAGTAATCGTCTCC | *prrf2* |
| pvdS100bp-1F | TGACAATCATTATCATTCAACATAATTTG | *pvdS* |
| pvdS100bp-1R | CATGGAAATCACCTTGCTGCG | *pvdS* |
| pvdS100bp-2F | CCGAAGAATTTCTCCCCTCCATC | *pvdS* |
| pvdS100bp-2R | TTGTCAATTACGAAGAAATAACCAATC | *pvdS* |
| pvdS100bp-3F | ACCGATTTCTAACTAGCTGATTCCTAAA | *pvdS* |
| pvdS100bp-3R | CTTCGGCGTTTCAGAAGGATGG | *pvdS* |
| pvdS qRT-PCR FWD Set 3 | GCAGATCACTTCGTCGTTC | *pvdS* |
| pvdS qRT-PCR REV Set 3 | CACGTTCAGGCCTTCTTC | *pvdS* |
| Prrf qRT-pcr F | AACTGGTCGCGAGATCAGC | *prrf1, 2* |
| Prrf qRT-pcr R | CCGTGATTAGCCTGATGAGGAG | *prrf1, 2* |
| prrH.for | ATTCGGCCGGAGACGACCGTT |  |
| prrH.rev | CGACCAGTTGGTGTAATAATAACTATT |  |
| oprF.for | GCGTTCGCAACATGAAGAAC | *prrf1, 2 control* |
| oprF.rev | CTTCTTGTTGCCGGTTTCGTA | *prrf1, 2 control* |
| prrF probe | TAAGCTGAGAGACCCACGCAGTCGG | *prrF* probe |
| prrH probe | CTGGCGATGGAATGAATGAGAACCG | *prrH* probe |
| oprF probe | CGGTGAGTACCATGACGTTCGTGGC | *oprF* probe |
| pscF-F-new 78bp | CATTCTTTCGCAGCTCACAG | pscEF promoter |
| pscF GSCont R93 | CCACGGTATCGAGGGTATTC | pscEF promoter |

**Literature Cited**

1. **Holloway BW.** 1955. Genetic recombination in *Pseudomonas aeruginosa*. J Gen Microbiol **13:**572-581.

2. **Lizewski SE, Lundberg DS, Schurr MJ.** 2002. The transcriptional regulator AlgR is essential for *Pseudomonas aeruginosa* pathogenesis. Infect Immun **70:**6083-6093.

3. **Wu W, Badrane H, Arora S, Baker HV, Jin S.** 2004. MucA-mediated coordination of type III secretion and alginate synthesis in *Pseudomonas aeruginosa*. J Bacteriol **186:**7575-7585.

4. **Okkotsu Y, Tieku P, Fitzsimmons LF, Churchill ME, Schurr MJ.** 2013. *Pseudomonas* *aeruginosa* AlgR phosphorylation modulates rhamnolipid production and motility. J Bacteriol **195:**5499-5515.

5. **Whitchurch CB, Erova TE, Emery JA, Sargent JL, Harris JM, Semmler AB, Young MD, Mattick JS, Wozniak DJ.** 2002. Phosphorylation of the *Pseudomonas aeruginosa* response regulator AlgR is essential for type IV fimbria-mediated twitching motility. J Bacteriol **184:**4544-4554.

6. **Cody WL, Pritchett CL, Jones AK, Carterson AJ, Jackson D, Frisk A, Wolfgang MC, Schurr MJ.** 2009. *Pseudomonas* *aeruginosa* AlgR Controls Cyanide Production in an AlgZ Dependent Manner. J Bacteriol **191:**2993-3002.

7. **Wilderman PJ, Sowa NA, FitzGerald DJ, FitzGerald PC, Gottesman S, Ochsner UA, Vasil ML.** 2004. Identification of tandem duplicate regulatory small RNAs in *Pseudomonas aeruginosa* involved in iron homeostasis. Proc Natl Acad Sci U S A **101:**9792-9797.

8. **Mathee K, Ciofu O, Sternberg C, Lindum PW, Campbell JI, Jensen P, Johnsen AH, Givskov M, Ohman DE, Molin S, Hoiby N, Kharazmi A.** 1999. Mucoid conversion of Pseudomonas aeruginosa by hydrogen peroxide: a mechanism for virulence activation in the cystic fibrosis lung. Microbiology **145 ( Pt 6):**1349-1357.

9. **Carterson AJ, Morici LA, Jackson DW, Frisk A, Lizewski SE, Jupiter R, Simpson K, Kunz DA, Davis SH, Schurr JR, Hassett DJ, Schurr MJ.** 2004. The transcriptional regulator AlgR controls cyanide production in *Pseudomonas aeruginosa*. J Bacteriol **186:**6837-6844.

10. **Ma S, Selvaraj U, Ohman DE, Quarless R, Hassett DJ, Wozniak DJ.** 1998. Phosphorylation-independent activity of the response regulators AlgB and AlgR in promoting alginate biosynthesis in mucoid *Pseudomonas aeruginosa*. J Bacteriol **180:**956-968.

11. **Okkotsu Y, Little AS, Schurr MJ.** 2014. The *Pseudomonas* *aeruginosa* AlgZR two-component system coordinates multiple phenotypes. Front Cell Infect Microbiol **4:**82.

12. **Qiu D, Damron FH, Mima T, Schweizer HP, Yu HD.** 2008. PBAD-Based Shuttle Vectors for Functional Analysis of Toxic and Highly Regulated Genes in *Pseudomonas* and *Burkholderia spp.* and Other Bacteria. Appl Environ Microbiol **74:**7422-7426.

13. **Figurski DH, Helinski DR.** 1979. Replication of an origin-containing derivative of plasmid RK2 dependent on a plasmid function provided *in trans*. Proc Natl Acad Sci USA **76:**1648-1652.

14. **Hoang TT, Karkhoff-Schweizer RR, Kutchma AJ, Schweizer HP.** 1998. A broad-host-range Flp-FRT recombination system for site-specific excision of chromosomally-located DNA sequences: application for isolation of unmarked *Pseudomonas aeruginosa* mutants. Gene **212:**77-86.

15. **Jones AK, Fulcher NB, Balzer GJ, Urbanowski ML, Pritchett CL, Schurr MJ, Yahr TL, Wolfgang MC.** 2010. Activation of the *Pseudomonas* *aeruginosa* AlgU regulon through *mucA* mutation inhibits cAMP/Vfr signaling. J Bacteriol **192:**5709-5717.

16. **Reinhart AA, Nguyen AT, Brewer LK, Bevere J, Jones JW, Kane MA, Damron FH, Barbier M, Oglesby-Sherrouse AG.** 2017. The *Pseudomonas aeruginosa* PrrF Small RNAs Regulate Iron Homeostasis during Acute Murine Lung Infection. Infect Immun **85**.

17. **Mohr CD, Hibler NS, Deretic V.** 1991. AlgR, a response regulator controlling mucoidy in *Pseudomonas aeruginosa*, binds to the FUS sites of the *algD* promoter located unusually far upstream from the mRNA start site. J Bacteriol **173:**5136-5143.
