# Supplementary material for: Pseudomonas aeruginosa AlgR Phosphorylation Status Differentially Regulates Pyocyanin and Pyoverdine Production
Source: mBio. 2018 Jan 30;9(1):e02318-17. doi: 10.1128/mBio.02318-17 (PMC5790918; doi:10.1128/mBio.02318-17)
Supplement: TEXT S1 [file mbo001183691s1.docx]

**MATERIALS AND METHODS**

**Bacterial strains, plasmids, genetic manipulations and growth conditions.** Bacterial strains, plasmids and oligonucleotides utilized are listed in Table S1. *P. aeruginosa* growth was at 37°C in LB-Miller or *Pseudomonas* Isolation Agar (PIA) (Difco). Chrome azul S (CAS) plates were prepared as previously described (1). *Escherichia coli* DH5α was maintained on LB-Miller (10g/l Tryptone, 5 gg/l yeast extract, 10g/l NaCl) agar plates with gentamicin (15 µg/ml) or ampicillin (100 µg/ml) as necessary. The induction plasmids pHERD30T-*algR*D54E and pHERD30T-*algR*D54N encoding AlgRD54E and AlgRD54N were described previously (2). PAO1 Δ*algZR* was created with pGEX18Gm Δ*algZR* (kind gift from Dr. Timothy Yahr) as previously described (3). PAO1*algZ*H175A was a site directed mutant generated with the overlapping oligos algZH175AF-HpyAV and algZH175AR-HpyAV, and the oligonucleotides algZ-1212FXbaI and algZ+1077RHindIII. This PCR product was digested with *Xba*I and *Hind*III and ligated into the suicide vector pEXG2, and allelic exchange was performed as described previously (3). M9 minimal media supplemented with 10 mM succinate or 10 mM glucose was used to examine carbon source differences in pyoverdine production (4).

**RNA isolation and preparation for Affymetrix GeneChip analysis.** Total RNA samples were prepared from three independent replicates of *P*. *aeruginosa* strains PAO1, PAO1 *algR*D54E and PAO1 *algR*D54N grown for 16 hrs in LB as previously described (5). Total RNA was extracted from cells using an RNeasy mini purification kit (Qiagen) per the manufacturer’s instructions. RNA quality and the presence of residual DNA were checked on an Agilent Bioanalyzer 2100 electrophoretic system pre- and post-DNase treatment. Total RNA was used for cDNA synthesis, fragmentation, and labeling according to the Affymetrix GeneChip *P*. *aeruginosa* genome array expression analysis protocol. Briefly, random hexamers (Invitrogen) were added (final concentration, 25 ng µl^-1^) to total RNA along with in vitro-transcribed *Bacillus subtilis* control spikes (as described in the Affymetrix GeneChip *P*. *aeruginosa* genome array expression analysis protocol). cDNA was synthesized using Superscript II (Invitrogen) according to the manufacturer’s instructions. RNA was removed by alkaline treatment and subsequent neutralization. The cDNA was purified with use of the QIAquick PCR purification kit (Qiagen) and fragmented by DNase I (0.6 U µg^-1^ of cDNA; Amersham) at 37°C for 10 min and then end labeled with biotin-ddUTP with use of the Enzo BioArray Terminal Labeling kit (Affymetrix) at 37°C for 60 min. Proper cDNA fragmentation and biotin labeling were determined by gel mobility shift assay with NeutrAvadin (Pierce) followed by electrophoresis through a 5% polyacrylamide gel and subsequent DNA staining with SYBR Green I (Roche).

**Microarray data analysis.** Microarray data were generated using Affymetrix protocols as previously described (5-7). Probe set summarization (.CHP) files were generated using the RMA (Robust Multi-chip Analysis) algorithm. Background-corrected perfect match intensities were computed for each perfect match cell on every GeneChip and base-2 logarithm of each background-corrected perfect match intensity was obtained. These background-corrected and log-transformed perfect match intensities were normalized using the quantile normalization method. Tukey's median polish was used to obtain estimates that serve as the log-scale expression measures associated with the particular probe set. These data were imported into Transcriptome Analysis Console (Affymetrix version 3.0.0) Transcripts that were absent under both control and experimental conditions were eliminated from further consideration. Signal estimates for each transcript cluster for each condition was calculated using Tukey's Bi-weight Average Algorithm. Statistical significance of signals between the control and experimental conditions (*P* < 0.05) for individual transcripts was determined using the *t* test. Finally, the mean value of the signal log ratios from each comparison file was calculated. Only those genes that met the above criteria and had a mean signal log ratio of greater than or equal to 1 for up-regulated transcripts and less than or equal to 1 for down-regulated transcripts were kept in the final list of genes. Signal log ratio values were converted from log2 and expressed as fold changes. The microarray data are available on the GEO (Gene Expression Omnibus) website at http://www.ncbi.nlm.nih.gov/projects/geo (GEO accession no. GSE97245).

**Total iron chelation capacity CAS plate assay.**  Detection of soluble iron chelating siderophores produced by surface adhered cells was performed utilizing CAS plate media, as previously described (1). Overnight cultures were spot inoculated onto CAS plates and allowed to grow for 48 hrs at 37°C. Zone of clearing around each colony was measured relative to their colony size and averaged across six replicates.

**Siderophore iron chelating equivalence assay.** The supernatant from cells grown for 16 hrs in LB broth was serially diluted in TBS in 96-well plates. Detection of siderophores was performed using a modified version of the CAS assay as described previously (1, 8). An equal volume of CAS dye solution containing 500 mM piperazine, 600 µM HDTMA, 150 µM chrome azurol S, and 15 µM FeCl_3_ at pH 5.6 was added to each well and allowed to incubate 24 hrs in the dark. The colorimetric assay was read at O.D._630_ and fit to a standard curve generated by known (0-80 µM) concentrations of the iron chelator ethylenediamine-N,N-diacetic acid (EDDA) and normalized to the O.D._600_ of the culture. The amount of siderophores produced was determined by the formula: Equivalent chelating capacity relative to PAO1= (OD_600nm_ PAO1/OD_600nm_ unknown) x µM EDDA unknown.

**Pyoverdine fluorescence assays.** The amount of pyoverdine produced by different *P.* aeruginosa strains in culture supernatants was measured as previously described (9). Culture supernatants were diluted into 96-well Nunc Fisher white-walled clear bottom plates and read at O.D._405_, which was the peak fluorescence emission of pyoverdine, and then normalized to the O.D._600_ of the culture. The amount of siderophores produced was quantified by the formula: O.D. _405_ supernatants relative to PAO1 = (OD_600nm_ PAO1/OD_600nm_ unknown) x O.D. _405_ unknown.

**Motility assays.** Subsurface twitching motility assays were performed as previously described (10). Overnight cultures were stab inoculated in the interstitial space between the plastic petri dish and the basal surface of LB-1.0% Bacto agar and were incubated for 48 hours at 37°C. Agar was removed and the plates stained with 0.5% crystal violet, and the diameter of the twitching zone was measured and plates were imaged using the ChemiDoc XRS system (Bio-Rad). Surface twitching motility assays were performed as previously described (11, 12). Overnight cultures were concentrated to 9 x 10^9^ cells/ml in morpholinepropanesulfonic acid (MOPS) buffer (10mM MOPS, 8mM MgSO4, [pH 7.6]). A 2.5µl volume of the suspension was spotted onto buffered twitching motility plates (10mM Tris, 8mM MgSO4, 1mM NaPO4, 0.5% glucose [pH 7.6], solidified with 1.5% Bacto agar) and was incubated at 37°C for 24 hours under 5% O_2_ (HERAcell 150; Thermo Scientific). The twitching zone was measured, and imaged using a digital camera and an Axiovert 25 inverted light microscope at 100 magnification (10 phase-contrast objective lens and 10 optical eyepiece) (Carl Zeiss).

**RT-qPCR.**  Reverse Transcription quantitative PCR (RT-qPCR) analysis was performed on total RNA extracted from 16 hr grown cultures of strains indicated in the figure legends, using RNeasy spin columns (Qiagen), and 1 µg of RNA was reverse transcribed to generate cDNA using Superscript II (Invitrogen). Real-time PCRs were conducted using Power SYBR Green (Life Technologies) and measured using a Bio-Rad CFX Connect real-time system with primer pairs shown in Table S1. Data were analyzed using CFX Manager 3.1 (Bio-Rad). Results were normalized to the reference gene *rpoD* expression.

For *prrf1, 2* RT-qPCR, RNA was extracted using the RNeasy Mini Kit (Qiagen) according to manufacturer's directions. A total of 50 ng/μl of RNA was used to generate cDNA with the ImPromII cDNA synthesis kit (Promega) as previously described (13), and cDNA was analyzed using the StepOnePlus instrument (Applied Biosystems) and TaqMan reagents (LifeTechnologies). Standard curves were produced for each primer-probe set listed in Table S1 by analyzing cDNA generated from serial dilutions of RNA and used to determine relative amounts of the RNAs as described previously (13). Relative RNA levels were then normalized to the levels of the oprF mRNA as previously described (13-16) .

**Western blot analysis of PvdS.** *P. aeruginosa* strains were grown in LB broth for 16 hrs. Cells were collected by centrifugation at 14,000 X g, resuspended in PBS, and lysed by sonication. Samples were normalized by total protein concentration to 10 µg as quantified by the Bradford protein assay (Bio-Rad), and were separated by SDS-PAGE on a 12% polyacrylamide gel and transferred to a polyvinylidene difluoride (PVDF) membrane (Bio-Rad). The membrane was blocked overnight in Tris Buffered Saline with 0.1% Tween (TBST) containing 5% skim milk and 1% bovine serum albumin (BSA). The membrane was probed with a 1:5000 dilution of monoclonal anti-PvdS antibody (kind gift of Dr. Michael L. Vasil), followed by a 1:20,000 dilution of horseradish peroxidase-conjugated goat anti-mouse antibody, and detected using the Clarity Western ECL substrate kit (Bio-Rad) and a Chemi-doc XRS system (Bio-Rad).

**Electrophoretic mobility shift assays (EMSA).** EMSA reactions were performed as done previously (2), utilizing either PCR generated 100-bp fragments or hybridized 25-bp oligonucleotides. Fragments of 100-bp were generated by PCR amplification using oligos PrrHF, PrrHRev, Prrf2For, and Prrf2Rev for *prrf1,2* and pvdS100bp-1F / pvdS100bp-1R, pvdS100bp-2F / pvdS100bp-2R, and pvdS100bp-3F / pvdS100bp-3R for *pvdS*, using PAO1 chromosomal DNA and Accuprime Taq DNA polymerase. Fragments of 25-bp were made by hybridizing two oligonucleotides (prrf2ABS-F and prrf2ABS-R, prrf2-neg1-F and prrf2-neg1-R, prrf2-neg1-F and prrf2-neg1-R) in 1x TE by heating to 95°C and annealed by cooling to room temperature. AlgR was incubated with DNA in our previously described reaction conditions (17), for 30 min. Reactions were run on non-denaturing 10% acrylamide gels. Resulting gels were visualized using the Sybr-Gold DNA stain (Invitrogen) and visualized by scanning on a Bio-Rad Molecular Imager FX Pro Plus phosphoimager.

**Pyocyanin production assay.** Pyocyanin production was measured as previously described (18, 19). Broth cultures were pelleted from strains PAO1, PAO1 *algR*D54E, and PAO1 *algR*D54N, and supernatants were transferred to fresh tubes. Pyocyanin was chloroform-extracted from supernatants using an equal volume of chloroform. The organic and aqueous layers were separated by centrifugation and the aqueous layer was discarded. HCl (0.2 N) was added to the chloroform to reduce pyocyanin and solubilize it into the aqueous acidic solution. The change in color of pyocyanin was measured by reading the absorbance of the aqueous layer at a wavelength of 520 nm. Measurements were standardized to total protein by lysing cell pellets and measuring protein amounts by Bradford protein assay (BioRad).

***Drosophila Melanogaster* oral feeding infection.** *Drosophila melanogaster* were maintained at 25˚C in sterile vials containing standard cornmeal-agar medium (Carolina Biological Supply Company) and netting. The fly population was allowed to reproduce to attain sufficient numbers of *Drosophila* for the experiments. Infections were performed as previously described (20, 21). Broth cultures of *P. aeruginosa* were grown to mid-log phase, collected by centrifugation, and adjusted to an OD_600_ of 2.0 using 5% sucrose. Strains were then spotted (120 µl) onto a sterile Whatman filter and allowed to dry for 30 minutes. The infection apparatus consisted of a sterile glass vial containing 5 mL of 5% sucrose agar with an inoculated Whatman filter placed on top. Male Canton S flies were aged 2-4 days and collected. The *Drosophila* were then starved for 2-3 hours in an empty glass vial prior to infection. Approximately, 8-12 *Drosophila* were placed into each infection apparatus containing *P. aeruginosa* and incubated at 25˚C in a humidity-controlled environment. *Drosophila* were anaesthetized using triethylamine during the sorting process, as previously described by Paternostro *et al.* (22). Approximately 25-30 *Drosophila* were observed for each infection. All strains were completed in triplicate. *Drosophila* populations were counted every 24 hours and subsequently recorded.

**Murine wound model.** 9-week old female C57BL/6J mice were used. Wounds were prepared as previously described (23). All mice had two back wounds inflicted using a 6 mm biopsy punch held open with 14 mm plastic O-rings sewn around the wound. Bacteria were topically applied to the wound and allowed to dry for 5 minutes. The wounds were subsequently dressed with Biobrane biological dressing, telfa pads, and their backs were covered in adhesive tegaderm. Wounds were harvested using an 8 mm biopsy punch, discarding the tegaderm and telfa but leaving the Biobrane biological dressing. This punch was subsequently either soaked in paraformaldehyde overnight to be used for histology, or suspended in 1ml PBS placed in a 2 ml tube with 1ml of 1 mm glass beads. The tissue was homogenized at 6,000 rpm in two 1-minute bursts in a MagNA Lyser (Roche) and the homogenate was serially diluted to determine CFU/ml.

**Murine acute pneumonia model.** P. aeruginosa strains were grown on PIA (Becton Dickinson) plates for 24 hrs at 37° C and suspended in 1X PBS. Eight Nine-week-old female BALB/c mice (Harlan Laboratories) were anesthetized by intraperitoneal injection of 0.25 ml of ketamine (6.7 mg/ml) and xylazine (1.3 mg/ml) in 0.9 % w/v saline for each *P. aeruginosa* strain tested. Anesthetized animals were placed on their backs and 10 µl inoculums were pipetted directly into each nostril (20 μl total). Bacterial doses were verified immediately after infection by serial dilution in 1x PBS + 1% BSA and plating on PIA. All animals were carefully observed for the duration of the trials according to University of Virginia Animal Care and Use Committee approved protocols. For statistical analysis of the survival curves, the Logrank test ran on GraphPad Prism v4.0 was used. Analysis of Mean time of death was performed using a one-way ANOVA with Bonferroni multiple comparison test ran on GraphPad Prism v4.0. To determine CFU/ml, animals were dissected and lungs were homogenized in 1ml PBS, plated at appropriated dilutions on PIA. Mice that survived to 70 hrs post infection were euthanized and CFU were determined as described above.

**Literature Cited**

1. **Schwyn B, Neilands JB.** 1987. Universal chemical assay for the detection and determination of siderophores. Anal Biochem **160:**47-56.

2. **Okkotsu Y, Tieku P, Fitzsimmons LF, Churchill ME, Schurr MJ.** 2013. *Pseudomonas* *aeruginosa* AlgR phosphorylation modulates rhamnolipid production and motility. J Bacteriol **195:**5499-5515.

3. **Hoang TT, Karkhoff-Schweizer RR, Kutchma AJ, Schweizer HP.** 1998. A broad-host-range Flp-FRT recombination system for site-specific excision of chromosomally-located DNA sequences: application for isolation of unmarked *Pseudomonas aeruginosa* mutants. Gene **212:**77-86.

4. **Miller JF.** 1972. Experiments in molecular genetics. Cold Spring Harbor Laboratory, New York.

5. **Morici LA, Carterson AJ, Wagner VE, Frisk A, Schurr JR, Zu Bentrup KH, Hassett DJ, Iglewski BH, Sauer K, Schurr MJ.** 2007. *Pseudomonas aeruginosa* AlgR Represses the Rhl Quorum-Sensing System in a Biofilm-Specific Manner. J Bacteriol **189:**7752-7764.

6. **Frisk A, Schurr JR, Wang G, Bertucci DC, Marrero L, Hwang SH, Hassett DJ, Schurr MJ.** 2004. Transcriptome analysis of *Pseudomonas aeruginosa* after interaction with human airway epithelial cells. Infect Immun **72:**5433-5438.

7. **Lizewski SE, Schurr JR, Jackson DW, Frisk A, Carterson AJ, Schurr MJ.** 2004. Identification of AlgR-regulated genes in *Pseudomonas* *aeruginosa* by use of microarray analysis. J Bacteriol **186:**5672-5684.

8. **Oram DM, Jacobson AD, Holmes RK.** 2006. Transcription of the contiguous *sigB,* *dtxR*, and *galE* genes in *Corynebacterium diphtheriae*: evidence for multiple transcripts and regulation by environmental factors. J Bacteriol **188:**2959-2973.

9. **Imperi F, Tiburzi F, Visca P.** 2009. Molecular basis of pyoverdine siderophore recycling in *Pseudomonas aeruginosa*. Proc Natl Acad Sci U S A **106:**20440-20445.

10. **Alm RA, Mattick JS.** 1995. Identification of a gene, pilV, required for type 4 fimbrial biogenesis in Pseudomonas aeruginosa, whose product possesses a pre-pilin-like leader sequence. Mol Microbiol **16:**485-496.

11. **Kearns DB, Robinson J, Shimkets LJ.** 2001. Pseudomonas aeruginosa exhibits directed twitching motility up phosphatidylethanolamine gradients. J Bacteriol **183:**763-767.

12. **Miller RM, Tomaras AP, Barker AP, Voelker DR, Chan ED, Vasil AI, Vasil ML.** 2008. Pseudomonas aeruginosa twitching motility-mediated chemotaxis towards phospholipids and fatty acids: specificity and metabolic requirements. J Bacteriol **190:**4038-4049.

13. **Reinhart AA, Powell DA, Nguyen AT, O'Neill M, Djapgne L, Wilks A, Ernst RK, Oglesby-Sherrouse AG.** 2015. The *prrF*-Encoded Small Regulatory RNAs Are Required for Iron Homeostasis and Virulence of *Pseudomonas aeruginosa*. Infect Immun **83:**863-875.

14. **Rao J, DiGiandomenico A, Unger J, Bao Y, Polanowska-Grabowska RK, Goldberg JB.** 2008. A novel oxidized low-density lipoprotein-binding protein from *Pseudomonas* *aeruginosa*. Microbiology **154:**654-665.

15. **Reinhart AA, Nguyen AT, Brewer LK, Bevere J, Jones JW, Kane MA, Damron FH, Barbier M, Oglesby-Sherrouse AG.** 2017. The *Pseudomonas aeruginosa* PrrF Small RNAs Regulate Iron Homeostasis during Acute Murine Lung Infection. Infect Immun **85**.

16. **Mourino S, Giardina BJ, Reyes-Caballero H, Wilks A.** 2016. Metabolite-driven Regulation of Heme Uptake by the Biliverdin IXbeta/delta-Selective Heme Oxygenase (HemO) of Pseudomonas aeruginosa. J Biol Chem **291:**20503-20515.

17. **Damron FH, Owings JP, Okkotsu Y, Varga JJ, Schurr JR, Goldberg JB, Schurr MJ, Yu HD.** 2012. Analysis of the *Pseudomonas aeruginosa* regulon controlled by the sensor kinase KinB and sigma factor RpoN. J Bacteriol doi:10.1128/JB.06105-11.

18. **Kurachi M.** 1958. Studies on the biosynthesis of pyocyanine. (II):Isolation and determination of pyocyanine. Bulletin of the Institute for Chemical Research **36:**174-187.

19. **Essar DW, Eberly L, Hadero A, Crawford IP.** 1990. Identification and characterization of genes for a second anthranilate synthase in *Pseudomonas aeruginosa:* interchangeability of the two anthranilate synthases and evolutionary implications. J Bacteriol **172:**884-900.

20. **Mulcahy H, Sibley CD, Surette MG, Lewenza S.** 2011. *Drosophila melanogaster* as an animal model for the study of *Pseudomonas aeruginosa* biofilm infections *in vivo*. PLoS Pathog **7:**e1002299.

21. **Sibley CD, Duan K, Fischer C, Parkins MD, Storey DG, Rabin HR, Surette MG.** 2008. Discerning the complexity of community interactions using a Drosophila model of polymicrobial infections. PLoS Pathog **4:**e1000184.

22. **Paternostro G, Vignola C, Bartsch DU, Omens JH, McCulloch AD, Reed JC.** 2001. Age-associated cardiac dysfunction in *Drosophila melanogaster*. Circ Res **88:**1053-1058.

23. **Guthrie KM, Agarwal A, Tackes DS, Johnson KW, Abbott NL, Murphy CJ, Czuprynski CJ, Kierski PR, Schurr MJ, McAnulty JF.** 2012. Antibacterial efficacy of silver-impregnated polyelectrolyte multilayers immobilized on a biological dressing in a murine wound infection model. Ann Surg **256:**371-377.
